# The TET3/GATA6 Axis Drives Lipid Metabolism and Therapeutic Vulnerabilities in Pancreatic Ductal Adenocarcinoma

**DOI:** 10.1002/advs.202501774

**Published:** 2025-06-26

**Authors:** Shuai Liu, Shaobo Kang, Na Lin, Wenqi Zhang, Shiwen Wang, Yuqing Ren, Xiaofan Ding, Jianxiu Yu, Ruiyu Xie

**Affiliations:** ^1^ Department of Biomedical Sciences Faculty of Health Sciences University of Macau Macau SAR 999078 China; ^2^ Ministry of Education Frontiers Science Center for Precision Oncology University of Macau Macau SAR 999078 China; ^3^ Department of Biochemistry and Molecular Cell Biology & Shanghai Key Laboratory of Tumor Microenvironment and Inflammation Shanghai Jiao Tong University School of Medicine Shanghai 200025 China

**Keywords:** epigenetic regulation, GATA6, lipid metabolic reprogramming, pancreatic ductal adenocarcinoma, TET3

## Abstract

Metabolic reprogramming is a hallmark of cancer, with dysregulated lipid metabolism contributing to tumor progression and therapy resistance. This study identifies the DNA demethylase TET3 as a key regulator of lipogenic metabolism in pancreatic ductal adenocarcinoma (PDAC). *TET3* expression is elevated in the lipogenic PDAC subtype and correlates with poor patient prognosis. Genetic ablation of *TET3* disrupts lipid homeostasis, alters the saturated‐to‐monounsaturated fatty acid ratio, and reduces proliferative capacity. Mechanistically, TET3 represses *GATA6* expression through recruitment of histone deacetylases (HDACs) to its promoter, leading to H3K27 deacetylation, independent of its catalytic activity. Loss of TET3 derepresses *GATA6*, which in turn suppresses lipogenic enzymes, such as stearoyl‐CoA desaturase (*SCD*) and acyl‐CoA synthetase long‐chain family member 3 (*ACSL3*), and sensitizes cells to ferroptosis. Notably, combined treatment with the HDAC inhibitor SAHA and the ferroptosis inducer Erastin significantly enhances gemcitabine‐induced cytotoxicity in lipogenic PDAC cells. These findings uncover a previously unrecognized non‐catalytic function of TET3 in sustaining lipid metabolic reprogramming in PDAC. Targeting the TET3/GATA6 axis in combination with ferroptosis and epigenetic modulators offers a promising strategy to overcome therapeutic resistance in aggressive pancreatic cancer.

## Graphical abstract

The DNA demethylase TET3 drives lipid metabolic reprogramming in pancreatic ductal adenocarcinoma via a non‐catalytic mechanism. TET3 recruits histone deacetylases to repress GATA6, sustaining lipogenic enzyme expression and ferroptosis resistance. Targeting the TET3/GATA6 axis in combination with ferroptosis and epigenetic modulators offers a promising strategy to overcome therapeutic resistance in aggressive pancreatic cancer.

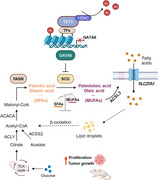

## 1 Introduction

Pancreatic ductal adenocarcinoma (PDAC) is a highly aggressive malignancy with a 5‐year survival rate of ≈13%.^[^ ^1^ ^]^ Due to late detection, only 15–20% of patients are diagnosed at a resectable stage.^[^ ^2^, ^3^ ^]^ Gemcitabine‐based chemotherapy remains the standard treatment, although modest improvements in survival are achieved. Based on genomic and transcriptomic profiling, PDAC has been classified into two major molecular subtypes: the basal‐like (or squamous) and classical (or progenitor).^[^ ^4^, ^5^, ^6^ ^]^ Moreover, recent advances in global metabolic profiling have identified three distinct metabolic phenotypes: the lipogenic, glycolytic, and slow‐proliferating subtypes, which are associated with different tumor behaviors and patient outcomes.^[^ ^7^, ^8^, ^9^, ^10^ ^]^ The lipogenic subtype, characterized by elevated lipid metabolites, is more susceptible to lipid biosynthesis inhibitors, whereas the glycolytic subtype enriches glycolytic intermediates and components of the pentose phosphate pathway (PPP). On the other hand, the slow‐proliferating subtype exhibits reduced levels of amino acids and carbohydrates, leading to a prolonged cell cycle and slower tumor growth compared to the lipogenic and glycolytic subtypes.^[^ ^8^ ^]^ These metabolic classifications underscore the importance of metabolic reprogramming in PDAC pathogenesis and highlight opportunities for metabolic‐targeted therapies.

One of the most profound metabolic alterations in cancer is the reprogramming of lipid metabolism, which supports rapid cell growth by providing essential macromolecules for membrane formation, maintaining membrane fluidity, and facilitating energy production.^[^ ^11^, ^12^ ^]^ Lipogenic remodeling is frequently observed across cancer types and involves enhanced *de novo* lipogenesis, fatty acid uptake, and altered cholesterol metabolism.^[^ ^13^, ^14^ ^]^ Key enzymes in this process, including ATP citrate lyase (ACLY), acetyl‐CoA synthetase (ACSS), fatty acid synthase (FASN), and stearoyl‐CoA desaturase (SCD), are often upregulated in tumors to meet increased biosynthetic demands.^[^ ^15^, ^16^ ^]^ Fatty acids, particularly monounsaturated fatty acids (MUFAs), support tumor growth, motility, and survival, whereas saturated fatty acids (SFAs) can induce lipotoxicity.^[^ ^17^, ^18^ ^]^ SCD, which catalyzes the desaturation of SFAs to Δ9‐MUFAs such as oleic and palmitoleic acid, plays a central role in maintaining lipid homeostasis and regulating ferroptosis sensitivity.^[^ ^18^, ^19^, ^20^ ^]^

Metabolic reprogramming in cancer is closely linked to epigenetic alterations, such as DNA methylation, which modulates gene expression and chromatin structure.^[^ ^21^, ^22^, ^23^ ^]^ DNA methylation is regulated by DNA methyltransferases (DNMTs)^[^ ^24^, ^25^ ^]^ and reversed by the ten‐eleven translocation (TET) family of dioxygenases.^[^ ^26^, ^27^ ^]^ Dysregulated DNA methylation can silence key metabolic genes, disrupting pathways including glycolysis, oxidative phosphorylation, and lipid metabolism.^[^ ^22^, ^23^ ^]^ For example, hypermethylation of the fatty acid transporter *SLC27A6* reduces cholesterol uptake and impairs metastatic potential in nasopharyngeal carcinoma.^[^ ^28^ ^]^ Conversely, hypomethylation of the *SCD* promoter has been associated with elevated expression in PDAC^[^ ^29^ ^]^ and other cancers, such as colorectal carcinoma and glioblastoma.^[^ ^30^ ^]^

Although accumulating evidence implicates DNA methylation in the regulation of cancer metabolism, the contribution of specific epigenetic modulators to lipid metabolic phenotypes in PDAC remains poorly understood. In this study, we identify TET3 as a critical regulator of lipid metabolism in PDAC. We show that TET3 expression is enriched in lipogenic PDAC tumors and correlates with elevated lipid biosynthetic activity. Genetic ablation of *TET3* impairs lipid droplet formation, disrupts the SFA/MUFA balance, and shifts metabolic programming toward a slow‐proliferating phenotype. Mechanistically, TET3 suppresses *GATA6* expression through histone deacetylation, independently of its catalytic dioxygenase function. Loss of TET3 increases GATA6 levels, which represses the expression of lipogenic enzymes including SCD and acyl‐CoA synthetase long‐chain family member 3 (ACSL3), thereby sensitizing cells to ferroptosis‐inducing agents. Furthermore, combinatorial treatment with the HDAC inhibitor SAHA and the ferroptosis inducer Erastin enhances gemcitabine efficacy in lipogenic PDAC cells. Together, these findings reveal a previously unrecognized TET3/GATA6 axis that drives lipid metabolic reprogramming in PDAC and suggest that targeting this pathway may offer a novel therapeutic strategy for metabolically aggressive disease.

## 2 Results

### 2.1 TET3 Positively Correlates with Lipogenic Features in Pancreatic Cancer

**Figure 1:**
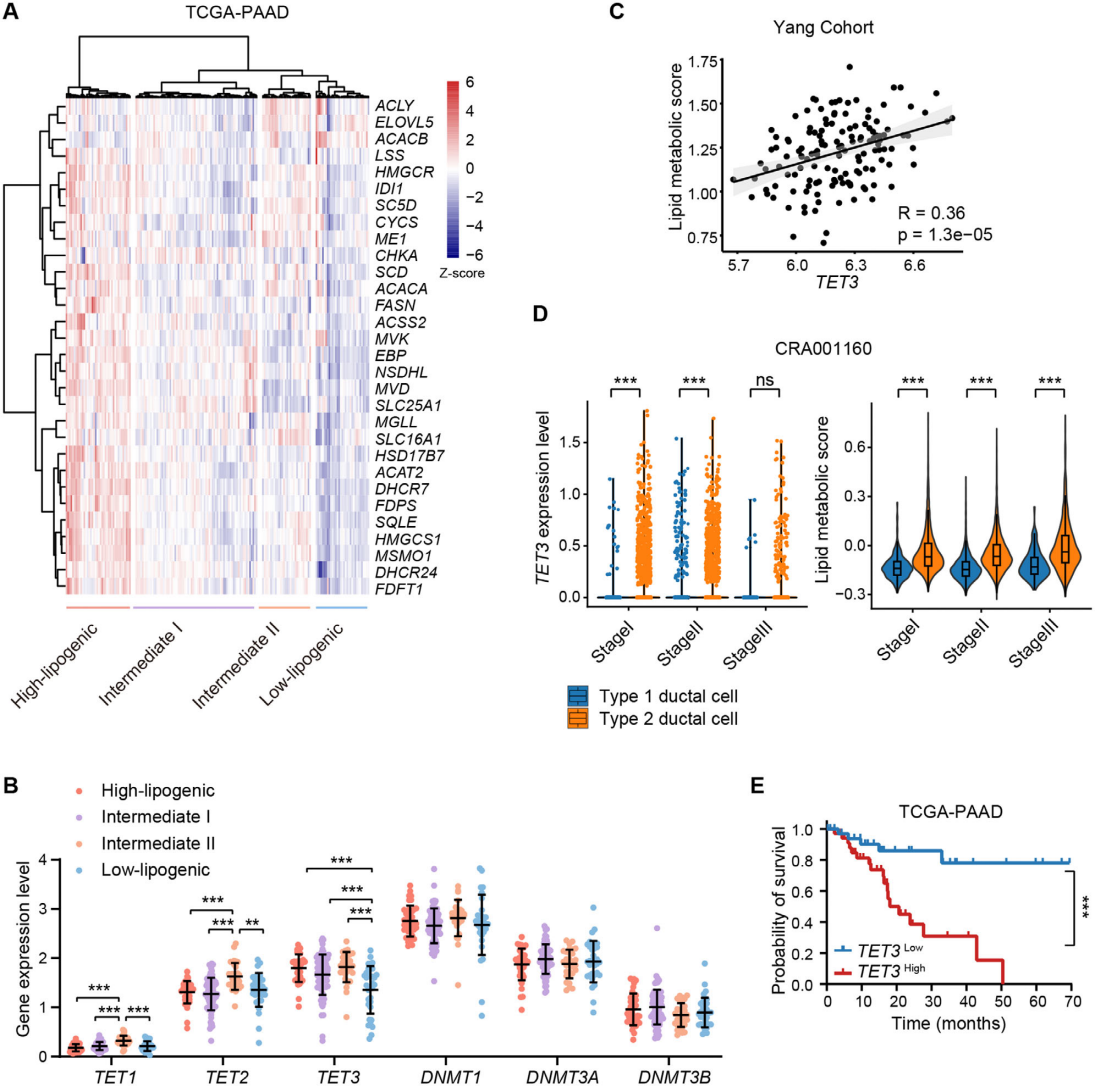
*TET3* positively correlates with lipogenic features in pancreatic cancer. A) Heatmap depicting the hierarchical clustering of TCGA‐PAAD patients (n = 178) into four lipogenic subgroups based on the expression of 30 previously reported lipogenic signature genes. B) mRNA expression levels (log₂(FPKM + 1)) of *DNMTs* and *TETs* across the four lipogenic groups: high‐lipogenic (n = 40), intermediate I (n = 75), intermediate II (n = 30), and low‐lipogenic (n = 33). C) Correlation between *TET3* expression and the lipogenic score in PDAC patients from the Yang cohort (GSE183795; n = 139). The lipogenic score was derived using single‐sample gene set enrichment analysis (ssGSEA) based on the same 30‐gene lipogenic signature. D) Violin plots showing the expression levels of *TET3* (left) and lipogenic signature scores (right) in type 1 and type 2 ductal cells previously identified in PDAC patients (CRA001160; n = 24).^[^ ^31^ ^]^ E) Kaplan‐Meier analysis of relapse‐free survival in TCGA‐PAAD patients with high (n = 34) or low (n = 35) *TET3* expression. Statistical significance was assessed by one‐way ANOVA (B), two‐tailed Wilcoxon test (D), or log‐rank Mantel‐Cox test (E). **p < 0.01, ***p < 0.001.

To investigate the role of DNA methylation regulators in lipid metabolism in pancreatic cancer, we analyzed the relationship between lipogenic signature genes and primary DNA methylation modifiers, DNA methyltransferases (DNMT1, DNMT3A, DNMT3B) and demethylases (TET1, TET2, TET3), using data from The Cancer Genome Atlas pancreatic adenocarcinoma (TCGA‐PAAD) cohort (n = 178 patients). Patients were categorized into four groups (high, intermediate I, intermediate II, and low‐lipogenic) based on the expression levels of 30 previously defined lipogenic signature genes (**Figure** **1**A).^[^ ^7^, ^8^ ^]^ Among the six DNA methylation regulators, *TET3* uniquely exhibited a strong and consistent positive correlation with lipogenic gene expression (Figure 1B). This association was not observed for other modifiers. Gene Set Enrichment Analysis (GSEA) further confirmed a significant enrichment of genes related to fatty acid metabolism in patients with high *TET3* expression (Figure S1A, Supporting Information), supporting a link between TET3 and lipogenic reprogramming in PDAC.

To validate these findings, we analyzed an independent PDAC patient dataset (Yang cohort; n = 139). Lipogenic features for each patient were quantified using lipogenic metabolic scores derived from the same 30‐gene lipogenic signature.^[^ ^7^, ^8^ ^]^ *TET3* expression again showed a significant positive correlation with lipogenic gene expression (Figure 1C). To determine whether this association was present in tumor epithelial cells, we examined a single‐cell RNA‐seq dataset of PDAC patients (GSE242230; n = 25). Clustering analysis revealed that *TET3* expression was primarily localized to epithelial cells and macrophages (Figure S1B, Supporting Information). Further subclustering of the epithelial cells based on lipogenic signature gene expression (Figure S1C, Supporting Information) revealed that cells with elevated lipogenic characteristics also exhibited higher *TET3* expression (Figure S1D, Supporting Information).

To assess the clinical relevance of TET3 and its associated lipid metabolic changes, we analyzed a second scRNA‐seq dataset (CRA001160) comprising 24 PDAC patients diagnosed at different stages (stage I: n = 9; stage II: n = 12; stage III: n = 3). *TET3* expression and lipid metabolic scores were evaluated in type 1 and type 2 ductal cells, previously characterized based on their malignant potential (Figure S1E, Supporting Information).^[^ ^31^ ^]^ Type 1 ductal cells resembled relatively normal ductal epithelium, while type 2 ductal cells displayed malignant features.^[^ ^31^ ^]^ Both *TET3* expression and lipid metabolic scores were markedly elevated in type 2 ductal cells across disease stages (Figure 1D), suggesting aberrant upregulation of *TET3* and lipogenesis‐related genes in the malignant population. Moreover, Kaplan‐Meier survival analysis indicated that high *TET3* expression was significantly associated with poorer survival (Figure 1E). In summary, these findings highlight a strong association between TET3 and lipogenic programs in PDAC, suggesting that TET3 may contribute to lipid metabolic reprogramming during malignant progression.

### 2.2 TET3 Depletion Alters Fatty Acid Metabolism in Pancreatic Cancer

**Figure 2:**
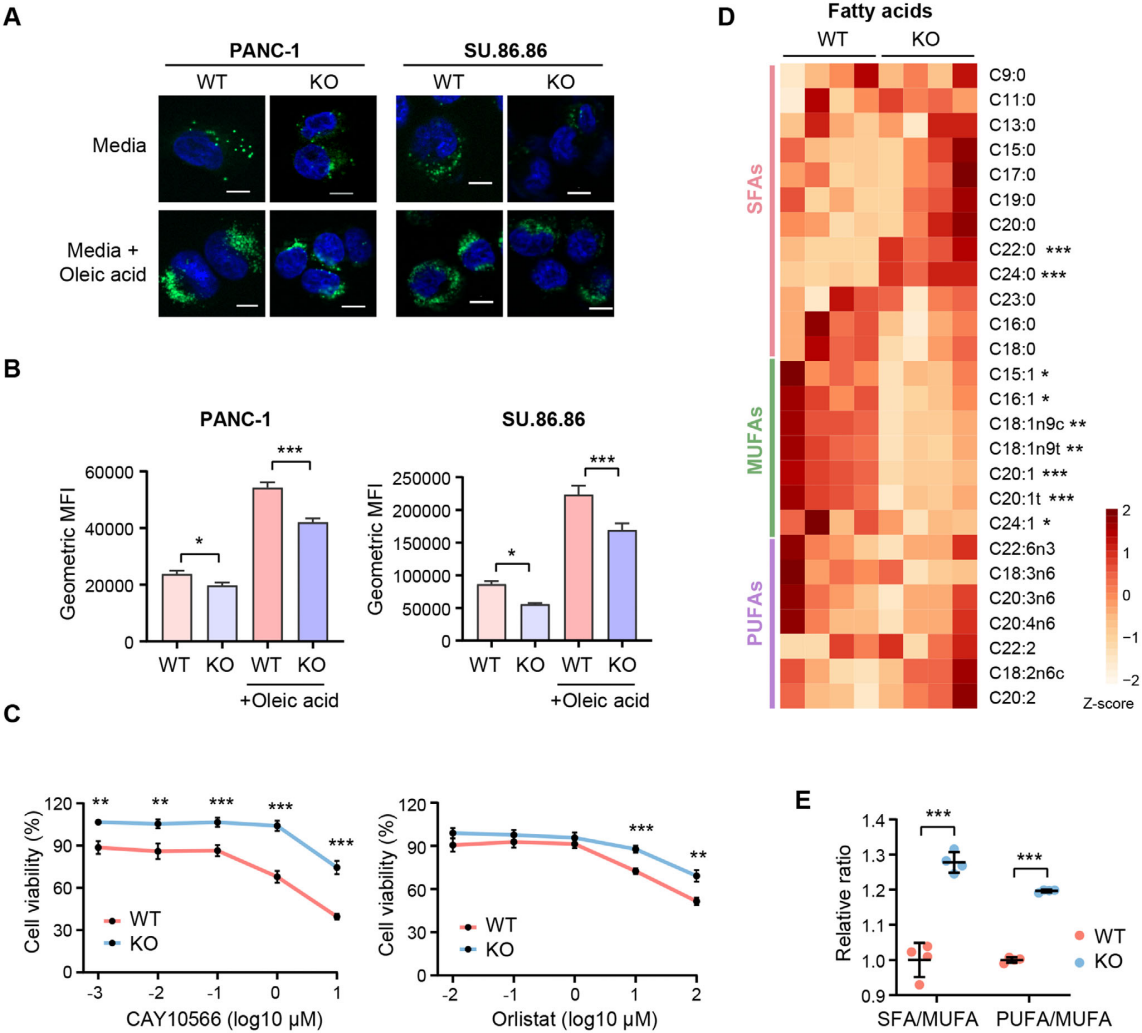
TET3 depletion alters fatty acid metabolism in pancreatic cancer. A) Representative images of lipid droplet staining in wild‐type (WT) and *TET3* knockout (KO) cells treated with or without oleic acid (200 µM) for 24 h. Lipid droplets were stained with BODIPY 493/503 (green), and nuclei with Hoechst 33342 (blue). Scale bars = 10 µm. B) Quantification of lipid accumulation by flow cytometry analysis of BODIPY 493/503 staining in WT and KO cells treated with or without oleic acid (200 µM) for 24 h (n = 3). C) Cell viability of WT and KO PANC‐1 cells treated with increasing concentrations of the SCD inhibitor CAY10566 (left) or the FASN inhibitor Orlistat (right) for 72 h, as measured by MTT assay (n = 3). D) Heatmap showing the relative abundance of fatty acids detected by GC‐MS‐based metabolomics in WT and KO PANC‐1 cells (n = 4). E) Quantification of saturated fatty acid (SFA) to monounsaturated fatty acid (MUFA) ratio and polyunsaturated fatty acid (PUFA) to MUFA ratio from metabolomics data (n = 4). Data represent mean ± SD. Statistical significance was determined using one‐way ANOVA (B) or two‐tailed unpaired *t*‐test (C, D, E). *p < 0.05, **p < 0.01, ***p < 0.001.

To investigate the role of TET3 in lipid metabolism, we generated CRISPR/Cas9‐mediated *TET3* knockout (KO) cell lines in two lipogenic PDAC cell lines, PANC‐1 and SU.86.86 (Figure S2A,B, Supporting Information).^[^ ^8^ ^]^ Fatty acids, essential precursors for lipid biosynthesis, are stored in lipid droplets as neutral lipids, primarily triglycerides and cholesterol esters, which serve as key reservoirs for energy and signaling.^[^ ^13^, ^18^ ^]^ To assess lipid droplet dynamics, we performed BODIPY 493/503 staining, a fluorescent dye that selectively labels neutral lipids.^[^ ^32^ ^]^ Both fluorescence microscopy and flow cytometry revealed a significant reduction in lipid droplet accumulation in TET3‐deficient cells compared to controls (**Figure** **2**A,B, Figure S2C, Supporting Information). Moreover, *TET3* knockout cells exhibited impaired fatty acid uptake and lipid droplet formation in response to oleic acid stimulation (Figure 2A,B, Figure S2C, Supporting Information). Nile Red staining further confirmed diminished lipid droplet content in TET3‐deficient cells (Figure S2D, Supporting Information). Collectively, these findings suggest that TET3 promotes both *de novo* lipogenesis and fatty acid uptake in pancreatic cancer cells.

As the lipogenic subtype of PDAC is often more susceptible to lipid synthesis inhibitors,^[^ ^8^ ^]^ we next tested the sensitivity of TET3‐deficient cells to pharmacologic blockade of key lipid synthesis enzymes. TET3‐depleted cells were significantly more resistant to the SCD inhibitor CAY10566 and the FASN inhibitor Orlistat compared to control cells (Figure 2C), indicating that TET3 supports lipid metabolic activity in PDAC.

To further elucidate the metabolic changes associated with TET3 loss, we conducted a targeted fatty acid metabolomics analysis in wild‐type and TET3_KO PANC‐1 cells. Principal component analysis (PCA) revealed clear separation in fatty acid profiles between the two groups (Figure S2E, Supporting Information). Specifically, MUFAs, including palmitoleic acid (C16:1) and oleic acid (C18:1), were significantly decreased in TET3‐deficient cells (Figure 2D), while SFAs, such as behenic acid (C22:0) and lignoceric acid (C24:0), were elevated. As a result, both SFA/MUFA and PUFA/MUFA ratios were markedly increased upon TET3 deletion (Figure 2E). Previous studies have linked elevated SFA/MUFA ratios to lipotoxicity and increased cell death.^[^ ^33^, ^34^, ^35^ ^]^ Consistently, we observed increased cell death in TET3‐deficient PANC‐1 cells (Figure S2F, Supporting Information). Together, these results identify TET3 as a key regulator of fatty acid composition and lipid homeostasis in pancreatic cancer cells.

### 2.3 TET3 Transcriptionally Regulates Genes Involved in Lipid Metabolism

**Figure 3:**
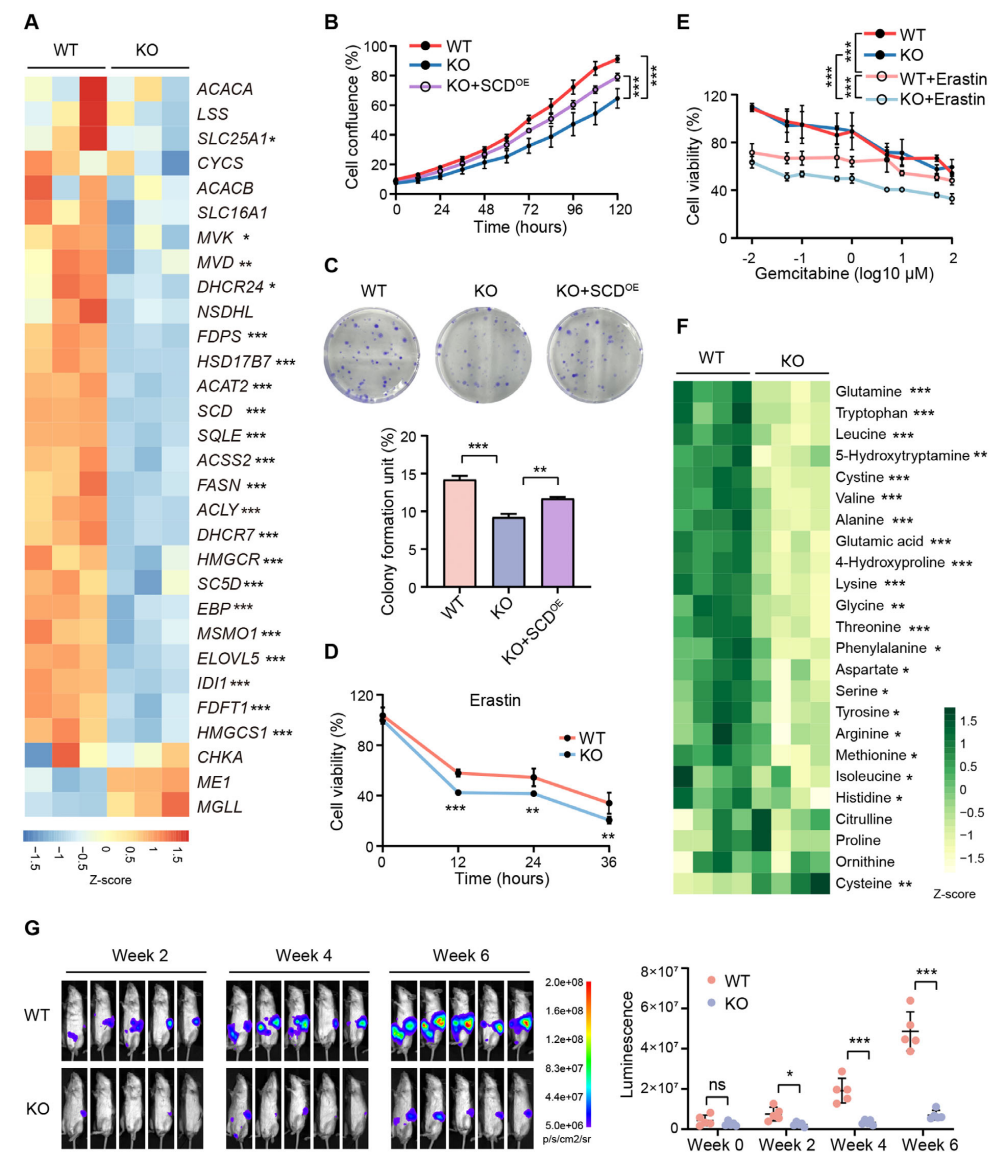
TET3 transcriptionally regulates lipid metabolic processes and tumor growth. A) Heatmap showing expression profiles of previously reported lipogenic signature genes in wild‐type (WT) and *TET3* knockout (KO) PANC‐1 cells (n = 3). Significantly downregulated genes are indicated with asterisks. Color key shows the Z‐score of normalized count values by DESeq2. B) Proliferation curves of WT, KO, and TET3‐deleted PANC‐1 cells with constitutive *SCD* overexpression (KO+SCD^OE^) measured by IncuCyte confluence analysis (n = 3). C) Colony formation assay of WT, KO, and KO+SCD^OE^ PANC‐1 cells cultured for 14 days (n = 3). D) Cell viability measured by MTT assay in WT and KO PANC‐1 cells treated with or without 3 µM Erastin (n = 3). E) Cell viability of WT and KO PANC‐1 cells treated with 3 µM Erastin combined with varying concentrations of gemcitabine for 24 h, assessed by MTT assay (n = 3). F) Heatmap representing amino acid profiles detected by GC‐MS metabolomics analysis in WT and KO PANC‐1 cells. Significantly changed metabolites are indicated with asterisks (n = 4). G) Bioluminescence imaging quantifying tumor burden in NOD‐SCID mice (n = 5) orthotopically transplanted with WT or KO PANC‐1 cells. Data are presented as mean ± SD. Statistical significance was determined by DESeq2 calculated FDR (A), repeated measures two‐way ANOVA (B, E, G), one‐way ANOVA (C), and two‐tailed unpaired *t*‐test (D, F). *p < 0.05, **p < 0.01, ***p < 0.001.

Fatty acids in cancer cells are primarily sourced from *de novo* synthesis and uptake from the surrounding tumor microenvironment.^[^ ^13^, ^18^ ^]^ To elucidate how TET3 promotes lipogenesis, we performed transcriptome profiling of wild‐type and TET3‐deficient PANC‐1 cells (Figure S3A, Supporting Information). Differential expression analysis identified 2,636 genes significantly altered upon TET3 deletion (Figure S3B, Table S1, Supporting Information), with enrichment in metabolic pathways such as cholesterol biosynthesis and fatty acid metabolism (Figure S3C, Supporting Information). Notably, 21 of the 30 previously defined lipogenic signature genes were significantly downregulated in TET3_KO cells compared to controls (**Figure** **3**A). Five representative genes involved in fatty acid metabolism, *SCD*, *FASN*, *FADS2*, *SLC27A1*, and *ACSL3*, were validated by real‐time quantitative PCR (RT‐qPCR), all of which showed reduced expression in both PANC‐1 and SU.86.86 TET3‐deficient cells (Figure S3D, Supporting Information).

Among the most significantly downregulated genes, *SCD* emerged as a potential target in TET3‐driven lipogenic reprogramming (Figure S3B, Supporting Information). SCD catalyzes the desaturation of SFAs into MUFAs,^[^ ^17^, ^18^ ^]^ and its inhibition is consistent with the elevated SFA/MUFA ratio observed in TET3_KO cells (Figure 2E). In line with the known role of SCD in promoting cancer cell proliferation,^[^ ^19^, ^20^ ^]^ TET3 loss significantly impaired cancer cell growth (Figure 3B) and colony formation (Figure 3C). Importantly, ectopic expression of *SCD* partially rescued the proliferation defects in TET3‐deficient cells (Figure 3B,C, Figure S3E, Supporting Information), indicating that SCD is a downstream effector of TET3‐mediated tumorigenesis.

ACSL3, another critical lipid metabolic enzyme, was also significantly downregulated in TET3_KO cells (Figure S3B,D, Supporting Information). Elevated expression of *SCD* and *ACSL3* has been associated with ferroptosis resistance, primarily by increasing the generation of MUFAs and promoting their incorporation into membrane phospholipids.^[^ ^36^, ^37^ ^]^ Given the concurrent reduction in MUFA levels and *ACSL3* expression upon TET3 deletion, we hypothesized that TET3‐deficient cells would be more susceptible to ferroptosis. Supporting this, TET3_KO cells exhibited increased sensitivity to the ferroptosis inducer Erastin compared to wild‐type cells (Figure 3D). Although Erastin alone sensitized pancreatic cancer cells to low‐dose gemcitabine, the combinatorial effect was particularly pronounced in TET3‐deficient cells (Figure 3E), revealing a potential therapeutic vulnerability. Taken together, these findings demonstrate that TET3 promotes lipid metabolic gene expression and contributes to ferroptosis resistance in PDAC, in part through the regulation of SCD and ACSL3, underscoring the potential of targeting TET3 in combination with ferroptosis‐inducing and chemotherapeutic agents.

### 2.4 Loss of TET3 Promotes a Slow‐Proliferating Phenotype in Pancreatic Cancer Cells

We next investigated whether TET3 loss induces a metabolic shift toward alternative PDAC subtypes. The glycolytic subtype is characterized by increased levels of glycolytic intermediates and PPP metabolites, whereas the slow‐proliferating subtype is defined by reduced amino acid availability and diminished tumor growth.^[^ ^8^ ^]^ To assess these possibilities, we performed targeted metabolomic profiling of amino acids (Figure S3F, Supporting Information) as well as key glycolytic and PPP metabolites (Figure S3G, Supporting Information). Notably, TET3‐deficient cells exhibited a significant reduction in several glycolytic and PPP metabolites, including glucose‐6‐phosphate, 3‐phosphoglycerate, and ribulose 5‐phosphate (Figure S3H, Supporting Information), suggesting that *TET3* knockout does not promote a compensatory glycolytic phenotype following impaired lipogenesis. In contrast, we observed a broad decrease in amino acid levels in TET3_KO cells (Figure 3F), indicative of a metabolic state consistent with the slow‐proliferating PDAC subtype. These metabolic alterations, coupled with the reduced proliferation and colony‐forming capacity of TET3‐deficient cells (Figure 3B,C), suggest that TET3 loss reprograms lipogenic PDAC cells toward a less proliferative state.

To determine whether TET3 loss affects tumor growth in vivo, we subcutaneously injected PANC‐1 cells with or without *TET3* expression into the flanks of immunodeficient nude mice. Tumor volume was monitored over an 8‐week period, and tumor weights were measured at endpoint. Tumors derived from TET3_KO cells exhibited significantly reduced growth and final mass compared to controls (Figure S3I,J, Supporting Information). To further evaluate the role of TET3 in orthotopic tumor growth, we transduced wild‐type and TET3_KO PANC‐1 cells with a lentiviral luciferase (*Luc2*) reporter and implanted them into the pancreata of NOD‐SCID mice. Bioluminescent imaging conducted at 2, 4, and 6 weeks post‐transplantation revealed a marked and sustained reduction in tumor growth in TET3‐deficient xenografts, as evidenced by significantly lower luminescent signal compared to controls (Figure 3G). These findings demonstrate that TET3 loss substantially impairs pancreatic cancer progression in vivo and reinforces its role in supporting tumor cell proliferation and metabolic activity.

### 2.5 TET3 Promotes the Lipogenic Program and Rapid Tumor Growth Independent of its Dioxygenase Activity

**Figure 4:**
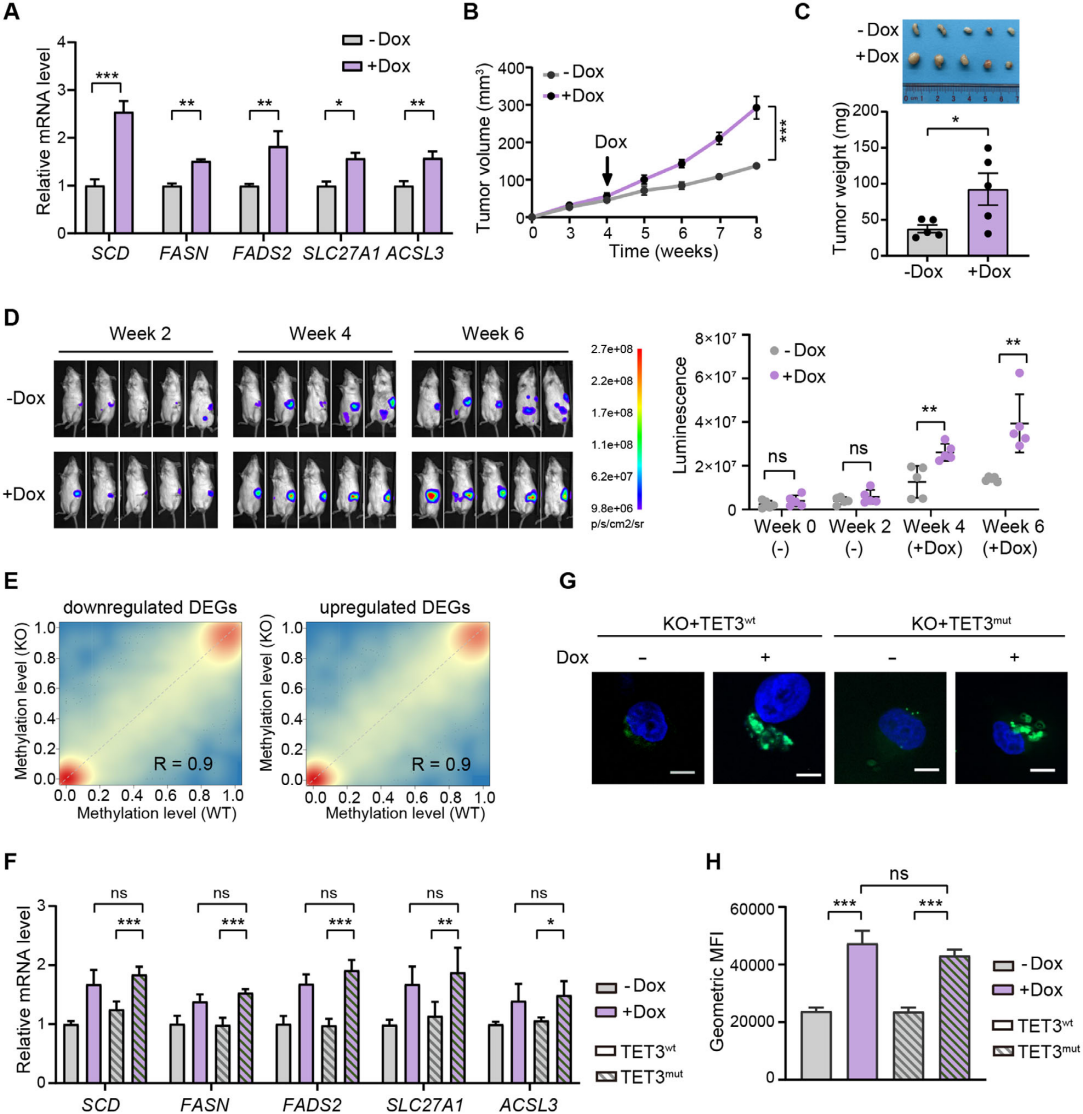
TET3 promotes the lipogenic program and rapid cancer growth independent of its enzymatic activity. A) RT‐qPCR analysis of lipid metabolic gene expression in TET3‐deleted PANC‐1 cells transduced with doxycycline‐inducible *TET3* cDNA, treated with or without doxycycline hyclate (Dox, 1 µg mL^−1^) (n = 3). B) Tumor growth curves of Nude mice subcutaneously transplanted with *TET3* knockout PANC‐1 cells receiving doxycycline (n = 5) or no doxycycline (n = 5). Doxycycline (0.5 mg mL^−1^) was administered in drinking water starting at week 4 post‐implantation to induce *TET3* expression. C) Representative images of subcutaneous xenografts at 8 weeks post‐implantation (top) and quantification of tumor weights (bottom). D) Bioluminescence analysis showing tumor growth in NOD‐SCID mice orthotopically transplanted with *TET3* knockout PANC‐1 cells, with (n = 5) or without (n = 5) doxycycline treatment. Doxycycline (0.5 mg mL^−1^) was provided in drinking water starting at week 2 post‐implantation to induce *TET3* expression. E) Scatter plots comparing DNA methylation levels at promoter regions of downregulated (left) and upregulated (right) differentially expressed genes (DEGs) between wild‐type (WT) and *TET3* knockout (KO) PANC‐1 cells. F) RT‐qPCR analysis of lipid metabolic gene expression in *TET3* knockout PANC‐1 cells transduced with doxycycline‐inducible wild‐type *TET3* (TET3^wt^, unfilled bars) or catalytically inactive mutant *TET3* (TET3^mut^, striped bars), treated with (purple) or without (gray) doxycycline (1 µg mL^−1^) (n = 3). G) Representative images of lipid droplet staining in *TET3* knockout PANC‐1 cells transduced with doxycycline‐inducible *TET3^wt^* or catalytically *TET3^mut^*, treated with or without doxycycline (1 µg mL^−1^). Lipid droplets stained with BODIPY 493/503 (green); nuclei stained with Hoechst 33342 (blue). Scale bars = 10 µm. H) Quantification of lipid droplets by flow cytometry (BODIPY 493/503) in *TET3* knockout PANC‐1 cells transduced with doxycycline‐inducible *TET3^wt^* (unfilled bars) or *TET3^mut^* (striped bars), treated with (purple) or without (gray) doxycycline (1 µg mL^−1^) (n = 3). Data are presented as mean ± SD. Statistical significance was determined by two‐tailed unpaired *t*‐test (A, C), repeated measures two‐way ANOVA (B, D), or one‐way ANOVA (F, H). *p < 0.05, **p < 0.01, ***p < 0.001.

To further validate the regulatory role of TET3 in pancreatic cancer, we reintroduced a doxycycline‐inducible human *TET3* cDNA into TET3‐deleted PANC‐1 cells. Upon doxycycline administration, TET3_KO cells exhibited robust re‐expression of *TET3* (Figure S4A,B, Supporting Information). RT‐qPCR analysis of key lipid metabolism genes, including *SCD*, *FASN*, *FADS2*, *SLC27A1*, and *ACSL3*, revealed a strong positive correlation between *TET3* expression and the upregulation of these targets (**Figure** **4**A). Importantly, restoration of *TET3* expression was sufficient to rescue the growth defects observed in TET3_KO cells both in vitro (Figure S4C, Supporting Information) and in vivo (Figure 4B–D), underscoring the critical role of TET3 in maintaining the lipogenic program and promoting rapid tumor growth in pancreatic cancer.

Given the canonical function of TET proteins as DNA demethylases, we next examined how TET3 depletion impacts the global DNA methylation landscape using whole‐genome bisulfite sequencing (WGBS). This analysis identified 15,225 differentially methylated regions (DMRs) between TET3_KO and control PANC‐1 cells, distributed across transcription start sites (TSS), gene bodies, and transcription end sites (TES) (Figure S4D, Supporting Information). Surprisingly, most of these regions exhibited decreased DNA methylation (hypo‐DMRs: 12,188), while only 3,037 showed increased methylation (hyper‐DMRs) (Figure S4E, Supporting Information). Moreover, methylation changes were not prominently enriched at promoters of differentially expressed genes (DEGs) (Figure 4E). Remarkably, key lipid metabolism genes downregulated in TET3_KO cells, such as *SCD*, did not exhibit hyper‐DMRs at their promoter or distal regulatory regions (Figure S4F, Supporting Information).

To directly test whether TET3 regulates lipid metabolism via its enzymatic activity, we expressed a doxycycline‐induced catalytically inactive mutant of *TET3* (*TET3^mut^*)^[^ ^38^ ^]^ in TET3_KO cells (Figure S4G,H, Supporting Information). Strikingly, expression of the *TET3^mut^* restored the expression of lipogenic genes (*SCD*, *FASN*, *FADS2*, *SLC27A1*, and *ACSL3*) to levels comparable to wild‐type *TET3* (*TET3^wt^*) (Figure 4F). Additionally, lipid droplet accumulation, an indicator of active lipid metabolism, was similarly rescued in TET3_KO cells upon expression of either *TET3^wt^* or *TET3^mut^* (Figure 4G,H, Figure S4I, Supporting Information). Together with the WGBS data, these findings demonstrate that TET3 supports lipid metabolic gene expression and tumor growth through a non‐catalytic mechanism, independent of its dioxygenase activity. This highlights a previously unexpected, enzyme‐independent role of TET3 in promoting lipogenic reprogramming and tumor progression in PDAC.

### 2.6 TET3 Transcriptionally Represses GATA6 via Histone Deacetylation

**Figure 5:**
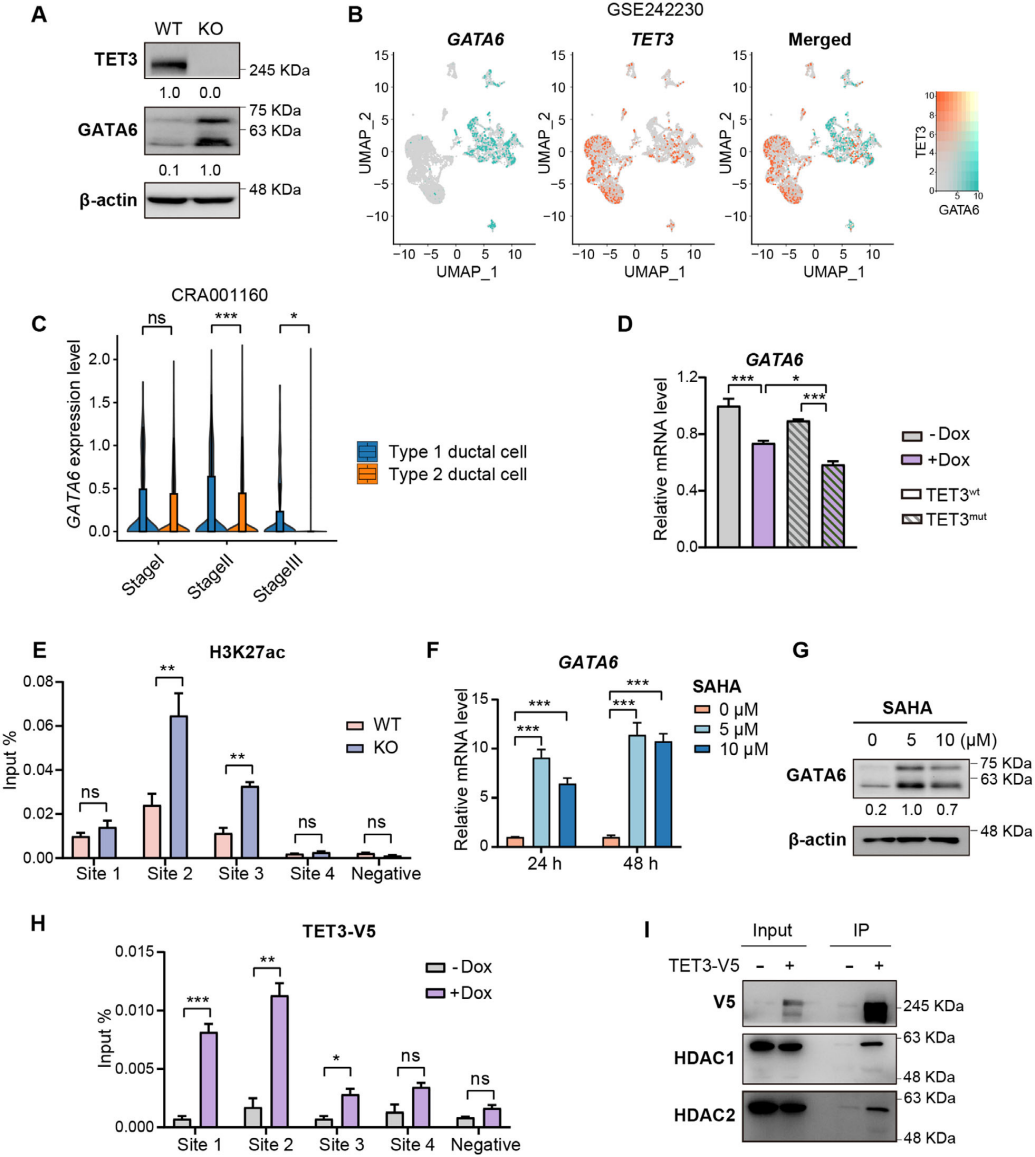
TET3 transcriptionally represses *GATA6* through histone deacetylation. A) Western blot analysis of GATA6 protein levels in wild‐type (WT) and *TET3* knockout (KO) PANC‐1 cells. B) UMAP visualization showing the expression distribution of *TET3* and *GATA6* in epithelial cells from scRNA‐seq of 25 PDAC patients (GSE242230). C) Violin plots showing the expression levels of *GATA6* in type 1 and type 2 ductal cells previously identified in PDAC patients (CRA001160; n = 24). D) RT‐qPCR analysis of *GATA6* mRNA in *TET3* knockout PANC‐1 cells transduced with doxycycline‐inducible wild‐type *TET3* (TET3^wt^, unfilled bars) or catalytically inactive mutant *TET3* (TET3^mut^, striped bars), treated with (purple) or without (gray) doxycycline (1 µg mL^−1^) (n = 3). E) ChIP‐qPCR assay of H3K27ac levels in WT and KO PANC‐1 cells (n = 3). Primers targeted regions upstream or downstream of the *GATA6* transcription start site, as indicated in Figure S5G, Supporting Information. F) RT‐qPCR measuring *GATA6* mRNA expression in wild‐type PANC‐1 cells treated with SAHA at 0, 5, or 10 µM for 24 or 48 h (n = 3). G) Western blot analysis of GATA6 protein levels in PANC‐1 cells treated with SAHA (0, 5, 10 µM) for 24 h. H) ChIP‐qPCR assay of V5 in PANC‐1 cells ectopically expressing V5‐tagged *TET3* (n = 3). qPCR primers are the same as those used in (E). I) Immunoprecipitation of V5‐tagged TET3 in PANC‐1 cells, followed by immunoblotting for HDAC1 and HDAC2 using an anti‐V5 antibody. Data represent mean ± SD. Statistical significance was determined by two‐tailed Wilcoxon test (C), one‐way ANOVA (D, F) or two‐tailed unpaired *t*‐test (E, H). *p < 0.05, **p < 0.01, ***p < 0.001.

Transcriptome profiling revealed a significant upregulation of *GATA6*, a critical regulator of pancreas differentiation, in TET3‐deficient cells (Figure S3B, Supporting Information). This increase was confirmed by RT‐qPCR and immunoblotting (**Figures** **5**A, S5A, Supporting Information). Notably, low *GATA6* expression is a hallmark of poorly differentiated, basal‐like PDAC subtypes.^[^ ^5^, ^39^, ^40^ ^]^ To explore a potential inverse relationship between *TET3* and *GATA6*, we analyzed *TET3* expression across PDAC patients with varing degrees of differentiation in the TCGA‐PAAD cohort. Tumors with low *TET3* expression were more frequently well‐differentiated, while high *TET3* expression was enriched in poorly differentiated cases (Figure S5B, Supporting Information). Consistent with this pattern, scRNA‐seq analyses from two independent PDAC datasets (GSE242230; n = 25 and GSE212966; n = 6) showed that *GATA6*‐expressing epithelial cells were largely excluded from TET3‐positive populations (Figure 5B, Figure S5C,D, Supporting Information). This natural exclusivity was further supported by a third independent dataset (CRA001160; n = 24), in which *GATA6* expression was markedly reduced in more malignant type 2 ductal cells from patients with advanced‐stage PDAC (Figure 5C), while *TET3* and lipogenic genes remained highly expressed (Figure 1D). Together, these data suggest that TET3 may promote pancreatic cancer progression, at least in part, by repressing *GATA6 expression*.

To test this hypothesis, we examined whether restoring *TET3* expression suppresses *GATA6* in TET3‐depleted cells. As expected, doxycycline‐induced expression of wild‐type *TET3* (*TET3^wt^*) significantly reduced *GATA6* transcript and protein levels (Figure 5D, Figure S5E, Supporting Information). Strikingly, the catalytically inactive mutant (TET3^mut^) repressed *GATA6* even more robustly than TET3^wt^ (Figure 5D), suggesting that this effect is independent of TET3's enzymatic activity. Supporting this, WGBS analysis revealed no appreciable changes in DNA methylation at the *GATA6* locus following TET3 deletion (Figure S5F, Supporting Information).

Given prior evidence that TET family proteins can interact with HDACs in various cellular contexts,^[^ ^41^, ^42^, ^43^ ^]^ we hypothesized that TET3 represses *GATA6* through histone deacetylation at its promoter. To assess this, we performed ChIP‐qPCR using an H3K27ac‐specific antibody in wild‐type and TET3_KO PANC‐1 cells. TET3‐deficient cells exhibited pronounced enrichment of H3K27ac at the *GATA6* promoter, indicating increased transcriptional activity, whereas control cells showed substantially lower signal at those sites (Figure 5E, Figure S5G, Supporting Information). To functionally validate the role of histone deacetylation, we treated wild‐type PANC‐1 cells with HDAC inhibitors, SAHA (Figure 5F,G) and Panobinostat (Figure S5H,I, Supporting Information), both of which led to increased *GATA6* expression, further supporting the role of HDACs in *GATA6 transcription*.

To determine whether TET3 directly engages the *GATA6* promoter, we performed ChIP‐qPCR in PANC‐1 cells overexpressing V5‐tagged *TET3*. Using the same primers employed in the H3K27ac ChIP assays (Figure 5E), we found TET3 occupied the same promoter regions marked by H3K27ac enrichment in TET3_KO cells (Figure 5H). Finally, co‐immunoprecipitation using a V5 antibody revealed that TET3 physically associates with HDAC1 and HDAC2, while no interaction was detected in control samples (Figure 5I). Together, these findings demonstrate that TET3 represses *GATA6* transcription via recruitment of HDACs and subsequent histone deacetylation at its promoter. This non‐catalytic epigenetic function of TET3 provides a mechanistic link between dedifferentiation and lipid metabolic reprogramming in pancreatic cancer.

### 2.7 GATA6 Suppresses Lipogenic Gene Expression and Tumor Growth

**Figure 6:**
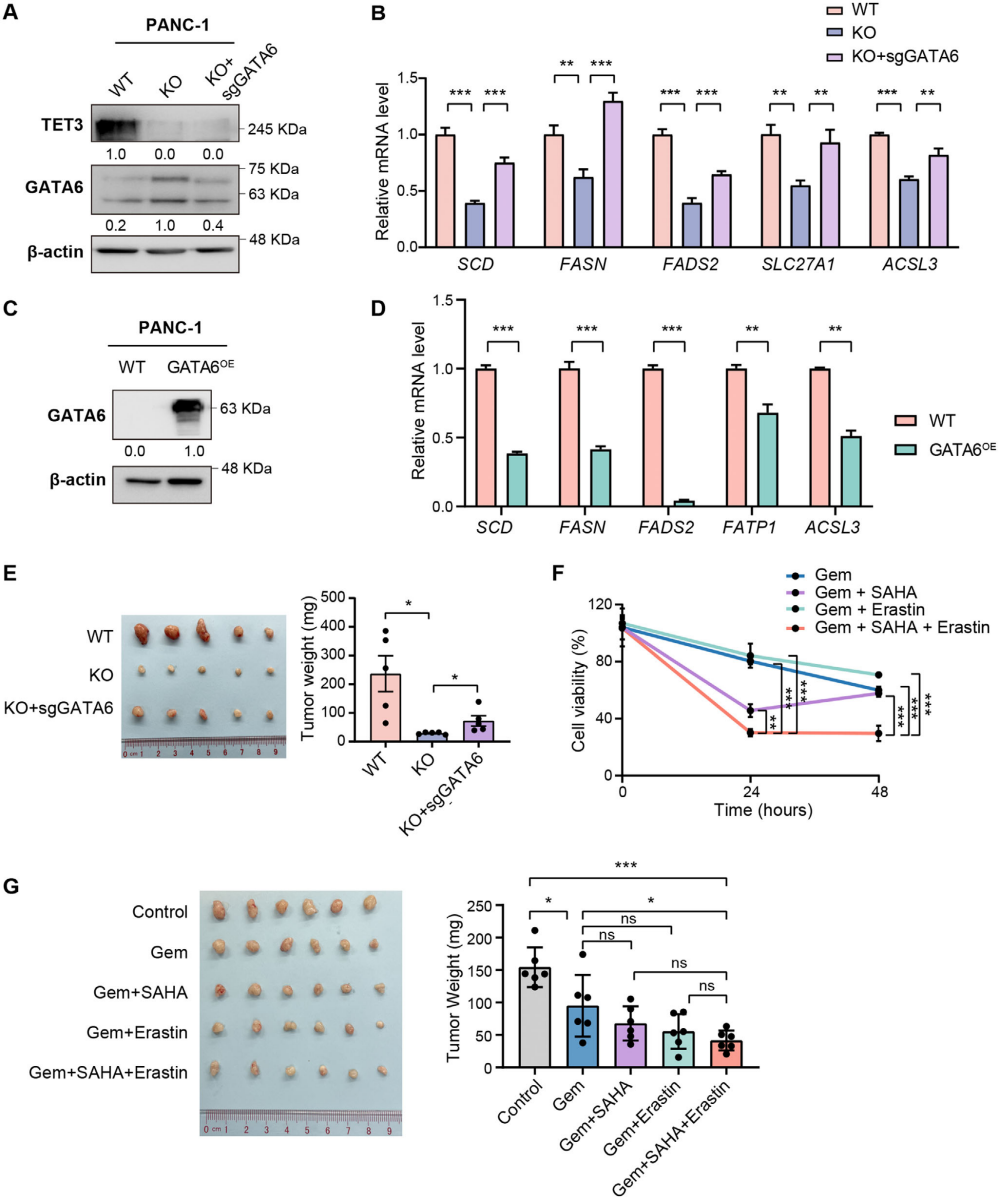
GATA6 suppresses lipogenic gene expression and limits tumor growth. A) Western blot showing knockout efficiency of *GATA6* in *TET3* knockout (KO) PANC‐1 cells. B) RT‐qPCR analysis of lipid metabolic gene expression in PANC‐1 cells with wild‐type (WT), KO, and *TET3*/*GATA6* double knockout (KO‐sgGATA6) (n = 3). C) Western blot showing overexpression efficiency of *GATA6* (GATA6^OE^) in wild‐type PANC‐1 cells. D) RT‐qPCR analysis of lipid metabolic gene expression in PANC‐1 cells with constitutive *GATA6* overexpression (GATA6^OE^) (n = 3). E) Representative images of subcutaneous xenograft tumors (left) and tumor weight at 8 weeks post‐implantation (right) for the indicated cell lines. Nude mice were transplanted with WT (n = 5), KO (n = 5), or KO‐sgGATA6 PANC‐1 cells (n = 5). F) Cell viability of wild‐type PANC‐1 cells treated for 24 or 48 h with gemcitabine (1 µM), gemcitabine + SAHA (5 µM), gemcitabine + Erastin (1 µM), or a triple combination of gemcitabine, SAHA, and Erastin (n = 3). G) Representative images of xenografts (left) and tumor weight at 7 weeks post‐implantation (right) following treatment beginning at week 4 with the indicated agents. Nude mice were transplanted with wild‐type PANC‐1 cells. Data represent mean ± SD. Statistical significance was determined by one‐way ANOVA (B, E, F, G) or two‐tailed unpaired *t*‐test (D). *p < 0.05, **p < 0.01, ***p < 0.001.

To investigate the functional role of *GATA6* in lipid metabolism and tumor growth, we first assessed its effect on the expression of fatty acid metabolism genes. CRISPR/Cas9‐mediated deletion of *GATA6* in TET3‐deficient cells reversed the suppression of key lipogenic genes, restoring their expression levels (**Figure** **6**A,B). Conversely, overexpression of *GATA6* in wild‐type PANC‐1 cells led to a marked reduction in the expression of these metabolic genes (Figure 6C,D), indicating that GATA6 negatively regulates lipogenesis in PDAC cells.

To evaluate the impact of GATA6 on tumor growth, we performed in vivo xenograft experiments. *GATA6* knockdown partially rescued the impaired tumor growth observed in TET3‐deficient cells (Figure 6E, Figure S6A, Supporting Information), supporting a model in which TET3 facilitates PDAC progression by suppressing *GATA6* and thereby maintaining a lipogenic gene expression program.

### 2.8 Therapeutic Targeting of the TET3/GATA6 Axis via Epigenetic and Metabolic Modulation

Given that TET3 represses *GATA6* through HDAC‐mediated histone deacetylation, we next explored whether HDAC inhibition could be used to counteract this regulatory mechanism. PANC‐1 cells were treated with low doses of the HDAC inhibitors SAHA (5 µM), the ferroptosis inducer Erastin (1 µM), and the chemotherapeutic agent gemcitabine (1 µM), either individually or in combination. Each agent alone had minimal impact on cell viability (Figure S6B, Supporting Information). However, simultaneous treatment with all three compounds led to >70% cell death within 24 h (Figure 6F). By contrast, dual combinations of SAHA‐gemcitabine or Erastin‐gemcitabine produced modest cytotoxic effects, highlighting the enhanced efficacy of the triple therapy.

To assess the translational relevance of this strategy, we tested the combination treatment in a subcutaneous xenograft model. While gemcitabine alone reduced tumor growth, the triple combination of gemcitabine, SAHA, and Erastin achieved a significantly greater reduction in tumor burden (Figure 6G). Together, these findings underscore the therapeutic potential of targeting the TET3/GATA6 axis using a combination approach that integrates epigenetic inhibition and ferroptosis induction. This strategy may enhance the efficacy of existing chemotherapy regimens and offer a novel avenue for treating advanced PDAC.

### 2.9 TET3 Promotes Invasive PDAC through Activation of the TGF‐β Signaling Pathway

**Figure 7:**
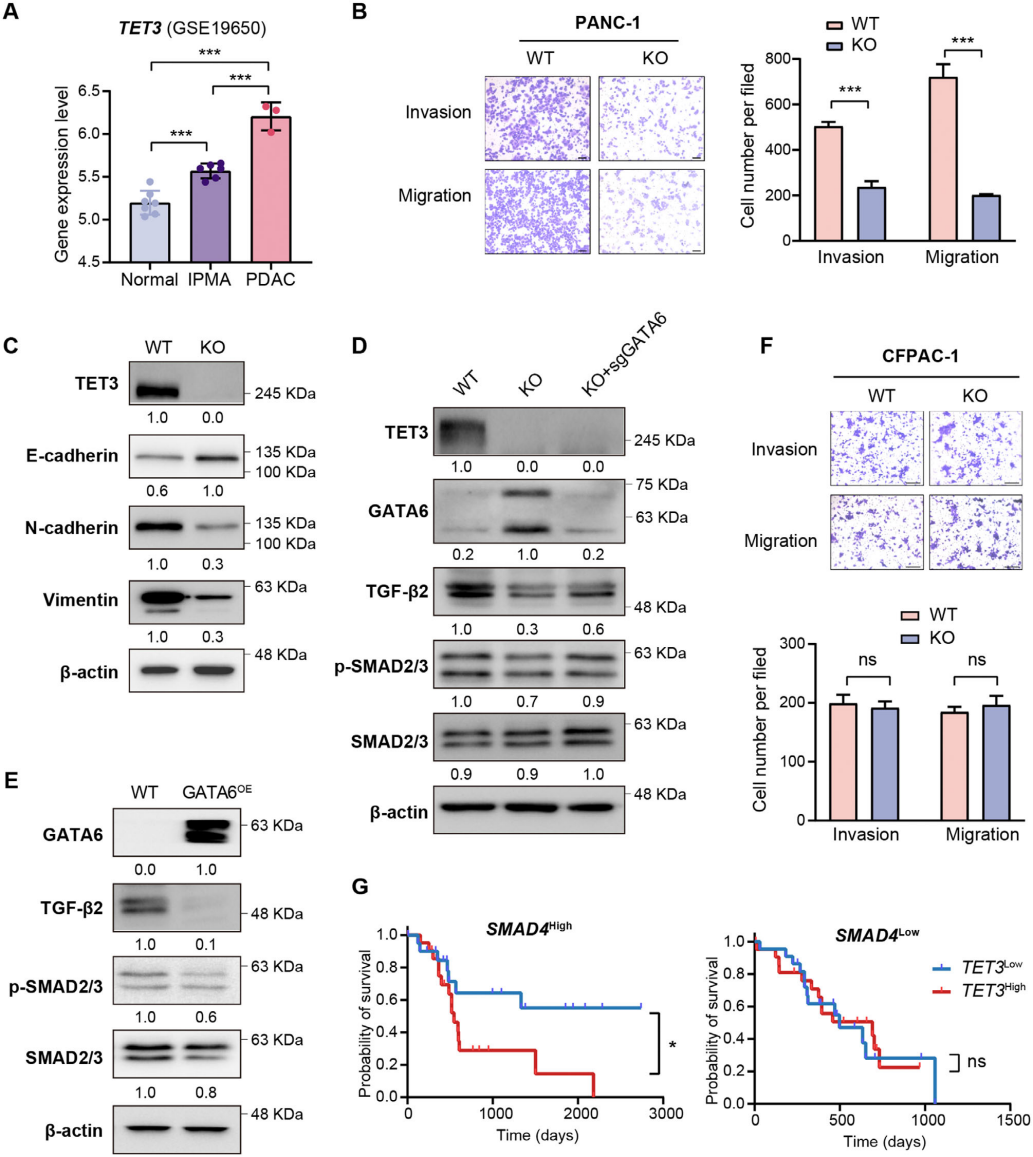
TET3 promotes invasive PDAC through activation of TGF‐β signaling pathway. A) mRNA expression levels of *TET3* in normal pancreatic tissues (n = 7), IPMA tissues (n = 6), and invasive PDAC tissues (n = 3) from GSE19650 (n = 16). B) Representative images and quantification of transwell invasion and migration assays in wild‐type (WT) and *TET3* knockout (KO) PANC‐1 cells (n = 3). Scale bars = 200 µm. C) Western blot analysis of epithelial‐mesenchymal transition (EMT) markers E‐cadherin, N‐cadherin, and vimentin in WT and KO PANC‐1 cells. D) Western blot analysis of TGF‐β pathway proteins in WT, KO, and *TET3*/*GATA6* double knockout (KO‐sgGATA6) PANC‐1 cells. E) Western blot analysis of TGF‐β signaling proteins in wild‐type PANC‐1 cells constitutively overexpressing *GATA6* (GATA6^OE^). F) Representative images and quantification of transwell invasion and migration assays in WT and KO in CFPAC‐1 cells (n = 3). G) Survival analysis of TCGA‐PAAD patients stratified by *SMAD4* expression (high: top 50%, n = 89; low: bottom 50%, n = 89) and further subdivided by *TET3* expression (high: top 25%, n = 22; low: bottom 25%, n = 22). Data are shown as mean ± SD. Statistical significance was assessed by one‐way ANOVA (A), two‐tailed unpaired *t*‐test (B, F), or log‐rank Mantel‐Cox test (G). *p < 0.05, **p < 0.01, ***p < 0.001.

To investigate the role of TET3 in PDAC progression, we analyzed a PDAC microarray dataset (GSE19650; n = 16 patients) and found a progressive increase in *TET3* expression from normal pancreatic tissue to premalignant intraductal papillary mucinous adenoma (IPMA), and ultimately to invasive PDAC (**Figure** **7**A). This trend suggested a potential involvement of TET3 in malignant transformation. Supporting this, analysis of a patient‐derived scRNA‐seq dataset (GSE197177; n = 7) revealed significantly elevated *TET3* expression in epithelial cells from hepatic metastases compared to primary PDAC tumors (Figure S7A,B), suggesting TET3 in metastatic progression.

Functionally, TET3 depletion markedly impaired the migratory and invasive capacities of PANC‐1 cells, as demonstrated by transwell assays (Figure 7B), with similar effects observed in the glycolytic PDAC cell line SW1990 (Figure S7C, Supporting Information). Reintroduction of *TET3* in knockout cells restored migration and invasion (Figure S7D, Supporting Information), reinforcing its pro‐invasive role. Mechanistically, TET3 loss increased expression of the epithelial marker E‐cadherin and decreased expression of the mesenchymal markers N‐cadherin and vimentin (Figure 7C, Figure S7E, Supporting Information), indicating that TET3 promotes epithelial‐to‐mesenchymal transition (EMT), a key step in tumor invasiveness.

To uncover the underlying mechanisms, we performed ATAC‐seq and identified 1,114 regions with increased (hyper), and 1,651 regions with decreased (hypo) chromatin accessibility in TET3‐deficient cells (Figure S7F, Supporting Information). Gene ontology analysis of differentially accessible regions (DARs) and differentially expressed genes (DEGs) revealed significant enrichment in the TGF‐β/SMAD signaling pathway (Tables S2 and S3, Supporting Information). Notably, both chromatin accessibility and transcript levels at the *TGFB2* locus were reduced in TET3‐deficient cells (Figures S3B and S7F, Supporting Information), accompanied by lower TGF‐β2 expression and decreased phosphorylation of SMAD2/3 (Figure 7D). These effects were partially reversed by *GATA6* knockout (Figure 7D). Conversely, overexpression of *GATA6* in wild‐type PANC‐1 cells suppressed TGF‐β2 expression and SMAD2/3 activation (Figure 7E), supporting a model in which TET3 activates TGF‐β signaling by repressing *GATA6*.

Since SMAD4 is a central effector of canonical TGF‐β signaling,^[^ ^44^ ^]^ we next examined its role in TET3‐driven invasion. TET3 loss had no detectable effect on migration or invasion in SMAD4‐negative CFPAC‐1 cells (Figure 7F, Figure S7G, Supporting Information). Moreover, SMAD4 deletion in TET3_KO PANC‐1 cells (Figure S7H, Supporting Information) abrogated the inhibitory phenotype of TET3 loss on invasion (Figure S7I, Supporting Information), suggesting that SMAD4 is necessary for the anti‐invasive effects observed upon TET3 depletion. Finally, Kaplan‐Meier survival analysis of TCGA‐PAAD data revealed that high TET3 expression correlated with poorer patient survival only in the subset of patients with high *SMAD4* expression, but not in those with low *SMAD4* levels (Figure 7G). Collectively, these results demonstrate that TET3 promotes PDAC cell invasion and EMT through activation of the SMAD4‐dependent TGF‐β pathway, in part by repressing GATA6.

## 3 Discussion

Dysregulated lipid metabolism is a hallmark of cancer and contributes to tumor progression and therapeutic resistance.^[^ ^16^ ^]^ In this study, we identify TET3 as a key regulator of lipid metabolism in PDAC. We demonstrate that *TET3* is highly expressed in PDAC cells with a lipogenic phenotype and is essential for maintaining lipid droplet accumulation and the conversion of SFAs to MUFAs (Figures 1 and 2). Mechanistically, TET3 upregulates the expression of *SCD*, a critical enzyme in this conversion process (Figure 3). While *SCD* overexpression has been previously associated with poor prognosis in multiple cancer types,^[^ ^45^, ^46^, ^47^ ^]^ our study is the first to establish its transcriptional regulation via TET3, thereby linking lipid metabolism to epigenetic control in PDAC.

Although TET3 has been implicated in both oncogenic and tumor‐suppressive roles in other cancers, our findings support its oncogenic function in PDAC. We observed a progressive increase in TET3 expression from normal pancreatic tissue to IPMA, invasive PDAC, and metastatic lesions (Figure 7). High TET3 expression also correlates with poor patient survival, suggesting its involvement in malignant progression.

TET3 functions here via a non‑catalytic mechanism. Despite its canonical role as a DNA demethylase, TET3 deletion did not lead to promoter hypermethylation of lipogenic genes; rather, whole‑genome bisulfite sequencing revealed a majority of hypomethylated DMRs in TET3‐deficient cells. This paradox likely reflects indirect or compensatory mechanisms, such as transcription‑factor occupancy blocking DNMTs,^[^ ^48^ ^]^ compensation by other TET family members,^[^ ^49^, ^50^, ^51^ ^]^ or secondary transcriptional changes, rather than the absence of TET3's catalytic activity. Importantly, a catalytically inactive TET3 mutant fully rescued lipogenic gene expression, confirming that TET3's promotion of lipid metabolism is dioxygenase‑independent. This aligns with prior studies demonstrating that TET family proteins can function in chromatin regulation independent of their enzymatic activity.^[^ ^52^, ^53^, ^54^, ^55^, ^56^ ^]^

Instead, TET3 directly represses the transcription factor *GATA6* through histone deacetylation (Figure 5), establishing a mechanistic basis for *GATA6* silencing in the lipogenic PDAC subtype. This TET3/GATA6 interaction adds to the growing evidence that GATA6 plays a context‐dependent tumor‐suppressive role in PDAC.^[^ ^39^, ^57^, ^58^, ^59^ ^]^ While TET2 has been shown to activate *GATA6* expression through 5‐hydroxymethylcytosine enrichment at its promoter,^[^ ^60^ ^]^ our study reveals that TET3 suppresses *GATA6* via HDAC recruitment, independent of DNA methylation changes. *GATA6* is highly expressed in classical PDAC and plays a pivotal role in suppressing EMT by promoting E‐cadherin and repressing vimentin.^[^ ^5^, ^39^, ^57^, ^58^ ^]^ Consistent with this, TET3 depletion restored *GATA6* expression, increased epithelial markers, and reduced invasive potential in PDAC cells (Figure 7), supporting a model in which TET3 drives EMT and invasion by silencing *GATA6*.

We further identified the TET3/GATA6 axis as a modulator of the TGF‐β signaling pathway, which is central to EMT, invasion, and metabolic reprogramming.^[^ ^61^, ^62^ ^]^ ATAC‐seq and gene expression analyses revealed that TET3 maintains chromatin accessibility at the *TGFB2* locus and promotes downstream SMAD2/3 phosphorylation (Figure 7). These effects are mediated by *GATA6* repression, at least in part, and require SMAD4, as *SMAD4* knockout abolishes the effects of TET3 depletion on cell invasion. Moreover, the link between high *TET3* expression and poor prognosis was evident only in patients with intact SMAD4, reinforcing the importance of this axis in TGF‐β‐driven malignancy.

Therapeutically, our findings suggest that disrupting the TET3/GATA6 pathway can reprogram lipid metabolism and sensitize PDAC cells to ferroptosis and chemotherapy. We show that HDAC inhibition with SAHA restores GATA6 expression, and when combined with the ferroptosis inducer Erastin and low‐dose gemcitabine, induces synergistic cytotoxicity in lipogenic PDAC cells (Figure 6). This highlights the potential of combinatorial epigenetic and metabolic therapies to overcome treatment resistance. Such strategies complement emerging approaches targeting the tumor microenvironment and metabolic vulnerabilities in solid tumors.^[^ ^63^, ^64^ ^]^

**Figure 8:**
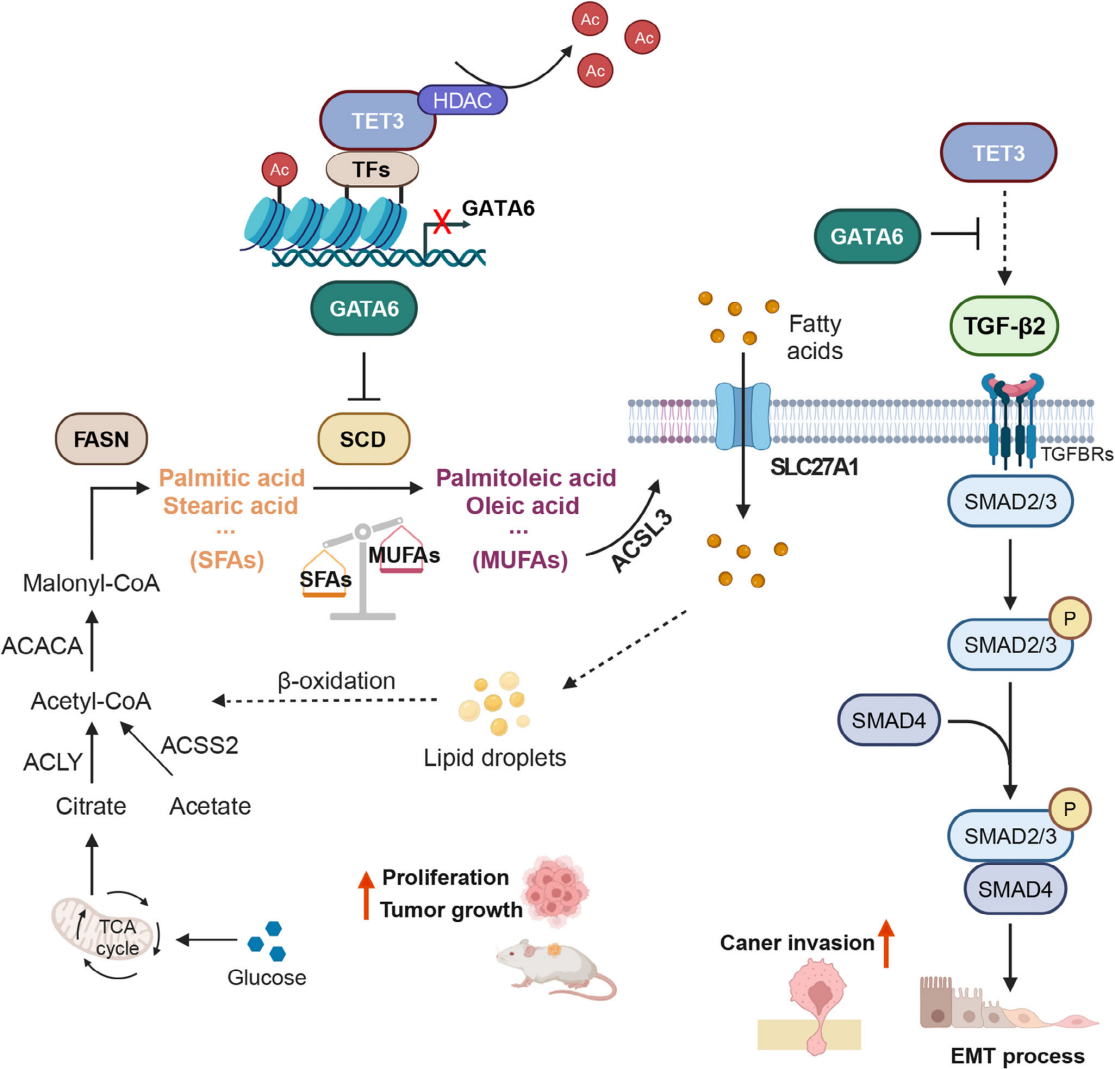
Schematic representation of the TET3/GATA6 axis in regulating lipogenic metabolism and promoting tumor growth and invasion in pancreatic cancer.

In summary, this study identifies a novel, non‐catalytic role for TET3 in driving PDAC progression through the repression of *GATA6*, promotion of lipogenesis, and activation of the TGF‐β signaling pathway (**Figure** **8**). Our findings establish the TET3/GATA6 axis as a critical regulator of both metabolic and invasive programs in PDAC and provide a rationale for therapeutic strategies targeting this pathway. Co‐targeting lipogenesis, ferroptosis, and epigenetic modulators may offer a promising approach to enhance the efficacy of existing therapies and address the metabolic adaptability of aggressive pancreatic tumors.

## 4 Experimental Section

### Ethics Approval

All animal experiments were approved by the University of Macau Animal Ethics Committee (UMARE‐0661‐2025). The Nude and NOD‐SCID mice were obtained from Charles River. Mice were housed in a pathogen‐free, temperature‐controlled environment, scheduled with a 12‐h light/dark cycle, and free access to standard mouse diet.

### Cell Lines and Cell Culture

Human PDAC cell lines CFPAC‐1 and PANC‐1 were obtained from the American Type Culture Collection (ATCC). Human PDAC cell line SW1990 was purchased from the Cell Bank of Type Culture Collection of the Chinese Academy of Sciences. PDAC cell line SU.86.86 was obtained from Prof. Qiang Chen at the Faculty of Health Sciences at the University of Macau. PANC‐1 and SW1990 were cultured in high‐glucose DMEM medium (Gibco, #12800017) supplemented with 10% FBS (Gibco, #26140079) and 1×Penicillin‐Streptomycin (Gibco, #15140163). SU.86.86 was cultured in RPMI 1640 medium (Gibco, #23400021) supplemented with 10% FBS and 1×Penicillin‐Streptomycin. CFPAC‐1 was cultured in IMDM medium (Gibco, #12200036) and 1×Penicillin‐Streptomycin supplemented with 10% FBS. All cell lines were maintained at 37 °C in a humidified incubator with 5% CO_2_.

### Lentiviral Production and Transduction

A mixture of 4 µg target plasmid, 3 µg psPAX2 (Addgene, #12260), and 2 µg VSV‐G (Addgene, #8454) was incubated with 27 µL polyethyleneimine (Polysciences, #23966‐1) in 800 µL serum‐free DMEM medium at room temperature for 20 min. The mixture was then transferred to 293T cells, and the virus was collected at 48 and 72 h using PEG‐8000 (Sigma‐Aldrich, #89510) concentration under 1,600 g for 60 min at 4 °C. Resuspended the pellets in cold PBS and aliquoted for storage at −80 °C. Cells were infected with the concentrated virus in the presence of 8 µg mL^−1^ polybrene for 48 h, followed by appropriate antibiotic selection.

### Establishment of Gene Expression Modified Cell Lines–Establishment of TET3 Knockout Cell Lines

CRISPR‐Cas9 genome editing was employed to deplete TET3 expression in PANC‐1, SU.86.86, SW1990, and CFPAC‐1 cells. TET3 sgRNA (Forward: CACCGCCGAAAAGG‐CCACCAGATCG, Reverse: AAACCGATCTGGTGGCCTTTTCGGC) was cloned into the lentiCRISPR v2 vector containing puromycin resistance (Addgene, #52961) and subsequently packaged into a lentiviral transduction system, followed by selection with 2 µg mL^−1^ puromycin (GeminiBio, #400‐128P) for 14 days.

### Establishment of GATA6 or SMAD4 Knockout Cell Lines in TET3‐Depleted Cells

The GATA6 sgRNA (Forward: CACCGAGTGGGCCAGCCAA‐CCACGC, Reverse: AAACGCGTGGTTGGCTGGCCCACTC) was cloned into the lentiGuide vector with hygromycin resistance (gifts from Prof. Kai Miao). SMAD4 sgRNA in the lentiGuide vector with hygromycin resistance was provided by Prof. Chuxia Deng. These constructs were then packaged into a lentiviral transduction system and introduced into the *TET3* knockout PANC‐1 cells, followed by selection with 400 µg mL^−1^ hygromycin B (Invitrogen, #10687010) for 14 days.

### Establishment of TET3 Rescued Cell Line

The human *TET3* (NM_001287491.2) with a sgRNA PAM silent mutation (C591A) was cloned into a doxycycline‐inducible PiggyBac vector (purchased from VectorBuilder, Guangzhou, China). The plasmid was transfected into *TET3* knockout cells using Lipofectamine 3000 Transfection Reagent (Invitrogen, #L3000008), following the manufacturer's instructions. After 3 days, 400 µg mL^−1^ hygromycin B was applied for selection over 2 weeks. Cancer cells were then treated with 1 µg mL^−1^ doxycycline hyclate (Sigma‐Aldrich, #D9891) for 3 days, with daily fresh drug replacement prior to experimental procedures.

### Establishment of TET3 Catalytically Inactive Cell Line

The mutagenesis is performed in the template plasmid using the human *TET3* sequence (NM_001287491.2) with a sgRNA PAM silent mutation (C591A) in a doxycycline‐inducible PiggyBac vector. The catalytically inactive sites (H1077Y, D1079A) were selected based on a previous report.^[^ ^38^ ^]^ The mutagenesis was generated by Q5 Site‐Directed Mutagenesis Kit (NEB, #E0552S) according to the manufacturer's instructions. In brief, exponential amplification was performed using Q5 Hot Start High‐Fidelity 2X Master Mix with specific primers targeting H1077Y and D1079A (Forward: aggcCCAGCA‐TAACCTCTACAATGGG, Reverse: tgtaGGCGTGGGCACAGAAGTC), followed by Kinase, Ligase & DpnI reaction for 5 min at RT. The product was then transformed into DH5α competent cells.

### Establishment of SCD Overexpression Cell Lines

*SCD* expression vector (NM_005063.5, purchased from General Biol, Chuzhou, China) was packaged into a lentiviral transduction system and used to infect *TET3* knockout PANC‐1 cells. Empty vector (Addgene, #120426) was transduced into wild‐type and TET3_KO cells as control groups. Following infection, cells were selected with 400 µg mL^−1^ hygromycin B for 14 days.

### Establishment of GATA6 Overexpression Cell Lines

GATA6 expression vector (Addgene, #120445) was packaged into a lentivirus transduction system and used to infect cancer cells. Empty vector (Addgene, #120426) was transduced into wild‐type cells as a control group. The cells were then selected under 400 µg mL^−1^ hygromycin B for 14 days.

### Lipid Droplet Analysis

For the microscope, 5 × 10^4^ cells were plated on gelatin‐coated micro cover glasses. Cells were treated with 200 µM oleic acid (Sigma‐Aldrich, #O3008) for 24 h to assess the uptake capability. Following a wash with PBS, cells were stained with 2 µM BODIPY 493/503 (Invitrogen, #D3922) for 15 min at 37 °C, or 5 µM Nile Red (Invitrogen, #N1142) for 8 min at 37 °C. The cells were then fixed with 4% PFA for 15 min at RT. Hoechst 33342 (1:5000) was applied for 10 min at RT. After mounting, images were captured using the Carl Zeiss APOTOME microscope. Scale bars represent 10 µM. For flow cytometry, 3 × 10^5^ cells were seeded into a 6‐well plate and treated with 200 µM oleic acid for 24 h to evaluate the uptake capability. After washing with PBS, cells were stained with 2 µM BODIPY 493/503 for 15 min at 37 °C. The cells were then harvested using trypsin and resuspended in 300 µL PBS. After filtration, the cells were analyzed using the Beckman CytoFLEX Flow Cytometer. Data and images were processed using FlowJo 10 software.

### MTT Assay

A total of 5,000 cells were plated into a 96‐well plate and incubated overnight. Drugs were added in a series of concentrations and incubated for 24–72 h at 37 °C in a humidified incubator with 5% CO_2_. Next, 10 µL MTT (Sigma‐Aldrich, #M5655) solution was added to a final concentration of 0.5 mg mL^−1^, and the cells were incubated for an additional 4 h. After removing the solution, 100 µL DMSO was added to dissolve the formazan crystals. The optical density (OD) absorbance was measured at 570 nm using the PerkinElmer Victor X5 Microplate Reader. Cell viability was calculated as OD (each concentration) / average OD (0 µM).

### Cell Death Assay

Annexin V/PI staining was performed to determine the percentage of apoptotic cells. 1 × 10^5^ cells were seeded into a 6‐well plate and cultured for 3–5 days. Cells were collected using TrypLE, washed with ice‐cold PBS, and resuspended in 100 µL Annexin V Binding Buffer (BioLegend, #422201) with 5 µL APC Annexin V (BioLegend, #640920) and 10 µL PI solution (BioLegend, #421301) for 15 min at RT. After washing with PBS and filtering, cells were analyzed using Beckman CytoFLEX Flow Cytometer. Data and images were processed with FlowJo 10 software.

### Incucyte Assay

For cell proliferation assay, 5,000 cells were seeded into a 96‐well plate and cultured for 5–6 days in the IncuCyte S3 Live‐Cell Analysis System. Images were captured every 3 h to calculate the cell confluence percentage.

### Colony Formation

500 cancer cells were seeded into a 6‐well plate and cultured for 14 days. Colonies were then fixed with 70% ethanol and stained with 0.5% crystal violet (Sigma‐Aldrich, #C0775). The colony formation unit (%) was calculated as (number of colonies / the number of seeded cells) ×100%.

### Transwell Assay

Matrigel (Corning, #356253) was diluted in serum‐free medium to a final concentration of 200 µg mL^−1^, and 100 µL was added to the center of the upper chamber insert with an 8 µm PET membrane (Corning, #354578) and incubated for 2 h at 37 °C. A suspension of 8 × 10^4^ cells in 200 µL serum‐free medium was seeded into the upper chamber, while 500 µL culture medium containing 10% FBS was added to the lower chamber. Cell invasion was assessed on the Matrigel‐coated membrane, whereas cell migration was assessed on the uncoated membrane. After 24–36 h culturing, cells were fixed with methanol and stained with 0.5% crystal violet (Sigma‐Aldrich, #C0775). Images were acquired using the Carl Zeiss APOTOME microscope, with scale bars set to 200 µM.

### Metabolomic Analysis

A total of 2 × 10^6^ cells were scraped and counted from a 10‐cm dish. After washing with ice‐cold PBS, the cell pellets were collected by centrifugation at 1.000 g for 10 min at 4 °C, followed by being soaked in liquid nitrogen for 10 min and stored at −80 °C for further analysis. Free fatty acids, amino acids, and energy metabolic products were quantified, and data were analyzed by Metware Biotechnology (Wuhan, China) using the Agilent 7890B‐7000D GC‐MS/MS platform. For sample preparation, 100 µL ultrapure water was added to resuspend the thawed cell pellet. Then, 50 µL cell suspension was combined with 75 µL HPLC‐grade methanol, 100 µL methyl tert‐butyl ether, and 25 µL 36% phosphoric acid solution. Following vertexing, soaking in liquid nitrogen, centrifugation, and drying, 300 µL of 15% boron trifluoride methanol was added, and the mixture was incubated at 60 °C for 30 min. After cooling to room temperature, 500 µL n‐hexane and 200 µL saturated sodium chloride were added, mixed thoroughly, and centrifuged at 12,000 rpm for 5 min at 4 °C. Then 100 µL of the n‐hexane layer was transferred for computer analysis. The sample derivatives were analyzed using the GC‐EI‐MS/MS system (Agilent 7890B).

### RNA‐Seq and Data Analysis

Total RNA was extracted using the MiniBEST Universal RNA Extraction Kit (Takara, #9767) according to the manufacturer's instructions. RNA quality assessment, library preparation, and sequencing were performed on the Novogene (Tianjin, China) platform using the HiSeq PE150 system. Raw FASTQ data were processed with Galaxy (https://usegalaxy.org/).^[^ ^65^ ^]^ Reads were trimmed using Trimmomatic v0.38.0 and aligned to the human reference genome (hg38) with HISAT2 v2.2.1 using default parameters. Annotation was completed with featureCounts v2.0.1 under default settings. The gene count matrix generated in Galaxy was imported into R v4.3.1 for differential expression analysis and data normalization using the DESeq2 v1.40.2 pipeline. DEGs were identified with |fold change| ≥ 1.2; false discovery rate (FDR) < 0.05. GSEA enrichment analysis was conducted with GSEA software v4.2.3, which was also used to generate image outputs.

### RNA Isolation and RT‐qPCR

Total RNA was extracted using Trizol reagent (Invitrogen, #15596026) following the manufacturer's instructions. cDNA was synthesized with the PrimeScript RT reagent kit (Takara, #RR037A). RT‐qPCR was performed in triplicate using the TB Green Premix Ex Taq (Takara, #RR420A), and the fluorescent signal was detected and analyzed with a Bio‐Rad CFX96 Touch Real‐Time PCR Detection System. Relative quantification was calculated using the 2^−ΔΔCt^ method, with TBP expression as the housekeeping gene. All primers used for RT‐qPCR are listed in Table S4 Supporting Information.

### WGBS and Data Analysis

Genomic DNA was isolated using the TIANamp Genomic DNA Kit (Tiangen, #DP304‐03). Library preparation and high‐throughput sequencing were conducted by Berry Genomics (Beijing, China). The WGBS data analysis was followed by the Kim et al. report.^[^ ^66^ ^]^ In brief, the raw reads were quality‐checked by FastQC (v0.11.4) and trimmed by TrimGalore (0.6.4).^[^ ^67^ ^]^ The remaining reads were then aligned against human reference hg38 using Bismark (v0.24.2)^[^ ^66^ ^]^ with paired‐end mode and did the deduplication by deduplicate_bismark. After that, methylation calling by bismark_methylation_extractor for generating CpG, CHG, and CHH context with ‘–CX –cytosine_report’ options. The CpG sites with a coverage of ≥5 were used for further analysis. DMRswere called by MethyKit (v0.99.2) with a window size of 1000 bp. To annotate for the genic and CpG annotations of the hyper‐DMRs and hypo‐DMRs, the R package annotatr (v1.28.0)^[^ ^68^ ^]^ was used. For the genes involved in the hyper‐DMRs and hypo‐DMRs, the R package ChIPseeker (v1.38.0) was used. To determine methylation level around the TSS, gene body, and TES, deeptools (v3.5.5)^[^ ^69^ ^]^ was implemented to calculate the methylation level across the above regions and visualize it.

### ATAC‐Seq and Data Analysis

ATAC‐seq libraries were prepared using the Active Motif's ATAC‐Seq Kit (#53150) following the manufacturer's instructions. The constructed libraries were then subjected to high‐throughput sequencing by Novogene (Tianjin, China). The ATAC‐seq data analysis began with the quality assessment of raw reads in FASTQ format using FastQC. Adapter sequences and low‐quality bases were removed with Cutadapt,^[^ ^70^ ^]^ and the cleaned reads were aligned to the hg38 human reference genome using Bowtie2 with default parameters.^[^ ^71^ ^]^ The resulting BAM files were sorted, and duplicate reads were marked using Picard tools. Chromatin accessibility was assessed by calling peaks with MACS2,^[^ ^72^ ^]^ using a *p*‐value cutoff of 0.01. These peaks were then annotated to the nearest genes using the ChIPseeker package.^[^ ^70^ ^]^ For quality control, the fraction of reads in peaks (FRiP) score was calculated. Differentially accessible regions between conditions were identified using DiffBind, with peaks exhibiting significant changes (adjusted *p*‐value < 0.05) considered differentially accessible. GREAT analysis (generated by http://great.stanford.edu/public/html/index.php ^[^ ^73^ ^]^) was used to perform biological processes annotation of loss of locus peaks.

### Animal Experiments

For subcutaneous transplantation, 2 × 10^6^ cancer cells suspended in 200 µL serum‐free DMEM were implanted into the right flank of 6‐8 weeks old male Nude mice. Subcutaneous tumor size was measured using digital calipers once or twice a week, with tumor volume calculated as *V* (mm^3^) = (*a* × *b* ^2^) / 2, where *a* is the longest diameter and *b* is the shortest diameter. For TET3 rescued groups, mice received drinking water containing 0.5 mg mL^−1^ doxycycline hyclate and 5% sucrose (Sigma‐Aldrich, #S0389), while the control group received water with only 5% sucrose; the water was refreshed every 3–4 days. For the drug treatment experiment, drug administration was initiated when tumor volumes reached ≈100 mm^3^, with treatments delivered via intraperitoneal injection twice weekly. The experimental groups were designed as follows: gemcitabine (Gem) alone (20 mg kg^−1^), gemcitabine (20 mg kg^−1^) + SAHA (50 mg kg^−1^) (Gem + SAHA), gemcitabine (20 mg kg^−1^) + Erastin (10 mg kg^−1^) (Gem + Erastin), and gemcitabine (20 mg kg^−1^) + SAHA (50 mg kg^−1^) + Erastin (10 mg kg^−1^) (Gem + SAHA + Erastin). Mice were euthanized at the endpoints, where tumors were harvested, and tumor weight was recorded.

For pancreas orthotopic transplantation, a suspension of 5 × 10^5^ cancer cells in 40 µL serum‐free DMEM mixed with 20 µL Matrigel was transplanted into the pancreatic tail of 6–8 weeks old male NOD‐SCID mice. Cells were labeled with Luciferase using a lentiviral transduction system (Addgene, #72485), followed by FACS sorting. For the TET3 rescued group, mice received drinking water containing 0.5 mg mL^−1^ doxycycline hyclate with 5% sucrose, while the control group received only 5% sucrose. Fresh drinking water with drugs was replenished every 3–4 days. Bioluminescence of orthotopic tumors was measured using the BLT Animal In Vivo Imaging System following an intraperitoneal injection of 100 µL D‐(‐)‐Luciferin potassium salt (15 mg mL^−1^ in saline) (Promega, #P1043). Bioluminescence values were analyzed with Phoenix software (BLT, v1.0.0). Mice were euthanized at the endpoints, where tumors were harvested, and GFP images were captured.

### Protein Extraction and Western Blot Analysis

Cells were scraped and lysed in ice‐cold lysis buffer (20 mM Tris‐HCl pH 7.5, 150 mM NaCl, 1 mM EDTA, 1% Triton X‐100, 5 mM 4‐nitrophenyl phosphate di (Tris) salt, 2 mM Na2VO4, 0.5% sodium deoxycholate, 1×Roche protease inhibitor cocktail) on ice for 20 min, followed by centrifugation at 14,000 g for 10 min at 4 °C. Protein concentration in the supernatant was determined using the DC Protein Assay (Bio‐Rad, #5000116). The supernatant was then mixed with 4× Laemmli sample buffer (250 mM Tris‐HCl pH 6.8, 40% glycerol, 8% SDS, 0.02% bromophenol blue) containing β‐mercaptoethanol, and boiled at 95 °C for 10 min for western blotting analyses. A total of 30‐60 µg protein per sample was separated on 8% or 10% acrylamide gel (Invitrogen, #HC2040) and subsequently transferred to the PVDF membrane (Millipore, #1620177). Membranes were blocked in 5% non‐fat milk for 1 h, followed by overnight incubation with the primary antibody at 4 °C. After washing with TBST, the membranes were incubated with the secondary antibody for 1 h. Images were captured using the Bio‐Rad ChemiDox MP Imaging System. Details of the antibodies used for western blotting are provided in Table S5, Supporting Information.

### Immunoprecipitation

Protein lysis was extracted as previously described in the protein extraction and western blot analysis section. A total of 4 mg supernatant was treated with 20 U nuclease (Takara, #2670A) at 4 °C for 30 min and then incubated with 4 µL mouse anti‐V5 antibody (Invitrogen, #R960‐25) overnight at 4 °C with gentle rocking, followed by incubation with 40 µL anti‐mouse Dynabeads (Invitrogen, #11201D) at 4 °C for 4 h. The protein‐antibody‐magnetic beads were washed three times with wash buffer A (20 mM Tris‐HCl pH 8, 0.3 M KCl, 10% glycerol, 1 mM EDTA, 1 mM DTT, 0.1% NP‐40, 1×Roche protease inhibitor cocktail) and two times with wash buffer B (20 mM Tris‐HCl pH 8, 0.1 M KCl, 10% glycerol, 1 mM EDTA, 1 mM DTT, 1×Roche protease inhibitor cocktail). Samples were resuspended with 30 µL protein lysis buffer and then mixed with 4× Laemmli sample buffer containing 10% β‐mercaptoethanol, followed by boiling at 95 °C for 10 min for western blotting analyses.

### ChIP Assay

≈1 × 10^7^ cells in a 10‐cm dish were crosslinked in 1% formaldehyde solution for 10 min. Cells were then scraped and lysed in 800 µL Lysis Buffer A (20 mM Tris‐HCl pH 7.5, 0.5% NP40, 50 mM KCl, 1×Roche protease inhibitor cocktail) for 10 min on ice. Following lysis, the sample was centrifuged at 2,000 rpm for 10 min at 4 °C, and the pellet was resuspended in 80 µL of Lysis Buffer B (20 mM Tris‐HCl pH 8, 1% SDS, 10 mM EDTA, 1×Roche protease inhibitor cocktail) and incubated on ice for 20 min. After incubation, 240 µL of Buffer C (20 mM Tris‐HCl pH 8, 2 mM EDTA, 1×Roche protease inhibitor cocktail) was added, and the lysate was sonicated using a Diagenode Bioruptor Plus for 10–14 min (30 s on, 30 s off) to generate DNA fragments of 200–800 bp. Cell debris was removed by centrifugation at 20,000 g for 10 min at 4 °C, the supernatant was diluted twofold, and 10 µL of supernatant was set aside as input. A total of 30–50 µg of the sheared chromatin was incubated overnight at 4 °C with 5 µg rabbit anti‐H3K27ac antibody, followed by a 4 h incubation with anti‐rabbit Dynabeads (Invitrogen, #11203D) at 4 °C. After reversing the crosslinks, the DNA was eluted and purified. Quantification of the resulting DNA was performed using the Bio‐Rad CFX96 Touch Real‐Time PCR Detection System. The primers used for RT‐qPCR analysis were listed in Table S4, Supporting Information.

### Analysis of the Publicly Available PDAC Studies–Expression Profile from TCGA

FPKM normalized gene expression data of GDC TCGA Pancreatic Cancer (TCGA‐PAAD) were downloaded from UCSC Xena (https://xena.ucsc.edu/).^[^ ^74^ ^]^ A total of 178 tumor samples were included for subsequent analysis. The information on pathological differentiation was obtained from the Pancreatic Expression Database (https://pancreasexpression.org/home/).^[^ ^75^ ^]^ GSEA analysis of TCGA‐PAAD patients was performed by GSEA software v4.2.3 based on raw counts data downloaded from the NCI GDC Data Portal. The lipogenic score was calculated using single‐sample gene set enrichment analysis (ssGSEA) implemented in the GSVA v2.0.7 R package, based on lipogenic signature genes from transcriptomic profiles.

### Expression Profile of Microarray from GEO

A total of 16 tissues of normal, pre‐malignant lesions, IPMA, and invasive PDAC were obtained from GSE19650.^[^ ^76^ ^]^ The microarray data were based on the GPL570 platform (Affymetrix Human Genome U133 Plus 2.0 Array). R package Affy v1.78.2 was used to convert the original CEL data into an expression matrix. A robust multi‐array average (RMA) approach was performed for background correction and normalization. A total of 139 human PDAC patients were obtained from GSE183795 (Yang cohort).^[^ ^77^ ^]^ The lipogenic score was calculated using ssGSEA implemented in the GSVA v2.0.7 R package.^[^ ^78^ ^]^

### Expression Profile of scRNA‐seq from GEO

Dataset CRA001160^[^ ^31^ ^]^ was employed to analyze the relationship between *TET3* expression level and lipogenic degree in PDAC ductal cells. GSE242230,^[^ ^79^ ^]^ GSE212966,^[^ ^80^ ^]^ and CRA001160^[^ ^31^ ^]^ were used to investigate the correlation between *TET3* and *GATA6* levels in PDAC tissues. Dataset GSE197177^[^ ^81^ ^]^ was employed to analyze the expression pattern of TET3 during PDAC metastasis. R package Seurat v4.3.0 was used to handle the gene‐cell matrix files downloaded from the GEO database. Cells expressing less than 200 or 500 genes and more than 15–25% mitochondrial reads were excluded from the downstream analysis, as in the previous reports.^[^ ^79^, ^80^, ^81^ ^]^ A total of 2,000 highly variable genes were selected and used to conduct PCA dimension reduction. The uniform manifold approximation and projection (UMAP) was performed. Epithelial ductal cells were included for subsequent analysis of gene expression. Lipid metabolic scores were calculated using the AddModuleScore from the R package Seurat.

### Survival Analysis

Relapse‐free survival analysis was performed by Kaplan‐Meier plotter (http://kmplot.com/analysis/).^[^ ^82^ ^]^ A total of 69 PDAC patients were used to calculate the relapse‐free survival according to TET3 expression level.

For the survival analysis in SMAD4 high and low patients, the survival and gene expression data were obtained from UCSC Xena GDC TCGA‐PAAD. The statistical analysis was calculated by Log‐rank (Mantel‐Cox) test using GraphPad Prism10.

### Statistical Analysis

Statistical analysis was carried out using IBM SPSS Statistics 27 to assess differences between experimental groups. At least three samples were used for statistical analysis. Data are presented as mean ± SD. Statistical significances were analyzed by two‐tailed unpaired t‐test for comparisons of two samples, one‐way ANOVA with Tukey's post hoc test for comparisons of three or more samples, and two‐way ANOVA test for bivariate comparisons. Statistics of nonparametric tests were analyzed by two‐tailed Wilcoxon test for comparisons of two samples. Pearson's chi‐squared test for categorical data. P<0.05 was considered to indicate a statistically significant difference.

## Conflict of Interest

The authors declare no conflict of interest.

## Author Contributions

R.X. conceived, directed, and oversaw the project. S.L. performed experiments and collected data. S.K., Y.R., and N.L. performed computational analysis for RNA‐seq, ChIP‐seq, ATAC‐seq, and WGBS. W.Z. and S.W. assisted with the animal studies. X.D. provided expert advice on bioinformatic analyses. J.Y. critically reviewed the manuscript. R.X. and S.L. wrote the manuscript; all other authors provided editorial advice.

## Supporting information



Supporting Information

Supporting Information

## Data Availability

The data that support the findings of this study are available from the corresponding author upon reasonable request.
