# Supplementary material for: The TET3/GATA6 Axis Drives Lipid Metabolism and Therapeutic Vulnerabilities in Pancreatic Ductal Adenocarcinoma
Source: Adv Sci (Weinh). 2025 Jun 26;12(37):e01774. doi: 10.1002/advs.202501774 (PMC12499388; doi:10.1002/advs.202501774)
Supplement: Supplementary file 1 — Supporting Information [file ADVS-12-e01774-s001.docx]

**Supporting Information**

**The TET3/GATA6 Axis Drives Lipid Metabolism and Therapeutic Vulnerabilities in Pancreatic Ductal Adenocarcinoma**

Shuai Liu^1^, Shaobo Kang^1^, Na Lin^1^, Wenqi Zhang^1^, Shiwen Wang^1^, Yuqing Ren^1^, Xiaofan Ding^1,2^, Jianxiu Yu^3^, Ruiyu Xie^1,2,^*

^1^ Department of Biomedical Sciences, Faculty of Health Sciences, University of Macau, Macau SAR 999078, China.

^2^ Ministry of Education Frontiers Science Center for Precision Oncology, University of Macau, Macau SAR 999078, China.

^3^ Department of Biochemistry and Molecular Cell Biology & Shanghai Key Laboratory of Tumor Microenvironment and Inflammation, Shanghai Jiao Tong University School of Medicine, Shanghai 200025, China.

* Co-corresponding author:

Ruiyu Xie, Ph.D.

Associate Professor, Associate Department Head

Department of Biomedical Sciences, University of Macau

Tel: +853 8822 4975; Fax: +853 8822 2902; Email: [RuiyuXie@um.edu.mo](mailto:RuiyuXie@um.edu.mo)

**This file includes:**

**Supplementary Figures 1-7 and supplementary Tables 4-6.**

**Supplementary Figures**

**Figure S1.** *TET3* positively correlates with lipogenic features in pancreatic cancer. A) Gene Set Enrichment Analysis (GSEA) of the fatty acid metabolism pathway in TCGA-PAAD patients grouped by *TET3* expression levels. Patients in the top and bottom 25^th^ percentiles were classified as *TET3*^high^ (n=44) and *TET*3^low^ (n=44), respectively. B) UMAP plot showing cell-type clustering within tumors from PDAC patients (GSE242230; n=25). Bright red indicates higher *TET3* expression levels across identified cell clusters. C) Heatmap displaying hierarchical clustering of epithelial ductal cells, identified in (B), into two subgroups based on the expression of 30 previously reported lipogenic signature genes. D) Comparison of *TET3* expression levels between the low-lipogenic and high-lipogenic epithelial subgroups defined in (C). E) UMAP plot showing type 1 and type 2 ductal cell populations within tumors from PDAC patients (CRA001160; n=24). Statistical significance was assessed using two-tailed Wilcoxon test (D). ***P<0.001.

**Figure S2.** TET3 depletion alters fatty acid metabolism in pancreatic cancer. A) Schematic showing the sgRNA target site within the *TET3* gene locus. B) Western blot confirming knockout efficiency of *TET3* in PANC-1 and SU.86.86 cells. C) Flow cytometric analysis of lipid droplet content using BODIPY 493/503 staining in wild-type (WT) and *TET3*-knockout (KO) cells, treated with or without 200 μM oleic acid (OA) for 24 hours. D) Representative fluorescence microscopy images showing lipid droplets (Nile Red, red) and nuclei (Hoechst 33342, blue) in WT and KO cells with or without oleic acid treatment (200 μM, 24 hours). E) Principal component analysis of fatty acid metabolomics profiles comparing WT and KO PANC-1 cells (n=4). F) Flow cytometric quantification of apoptosis using annexin V/PI staining in WT and KO PANC-1 cells (n=9). Scale bars = 10 μm. Data are presented as mean ± SD. Statistical significance was determined by two-tailed unpaired t-test (F). ***P<0.001.

**Figure S3.** TET3 regulates lipid metabolism and promotes tumor growth in pancreatic cancer. A) Principal component analysis (PCA) showing transcriptomic differences between wild-type (WT) and *TET3*-knockout (KO) PANC-1 cells (n=3). B) Volcano plot of differentially expressed genes (DEGs) comparing KO to WT PANC-1 cells. Upregulated genes are highlighted in red; downregulated genes in blue. The x-axis indicates log_2_ fold change; the y-axis represents the square root of -log_10_ FDR (|fold change|≥1.2; FDR<0.05). C) Gene Set Enrichment Analysis plots showing enrichment of cholesterol homeostasis and fatty acid biosynthetic processes in DEGs. Normalized enrichment scores (NES) and p-values are shown. D) RT-qPCR analysis of lipid metabolism-related genes in WT and KO cells (n=6). E) Western blot verifying *SCD* overexpression in *TET3*-knockout PDAC-1 cells transduced with lentiviral *SCD* cDNA (KO+SCD^OE^). F) PCA of amino acid metabolomics profiles comparing WT and KO PANC-1 cells (n=4). G) PCA of glycolysis metabolomics profiles in WT and KO PANC-1 cells (n=4). H) Heatmap of glucose metabolism intermediates measured by GC-MS. Metabolites with significant changes are indicated with asterisks (n=4). I) Tumor growth curves in nude mice subcutaneously implanted with WT or KO PANC-1 cells (n=5). J) Representative images of tumors at 8 weeks post-implantation (top) and corresponding tumor weights (bottom). Data are presented as mean ± SD. Statistical significance was assessed using two-tailed unpaired t-test (D, H, J) and repeated measures two-way ANOVA (I). *P<0.05, **P<0.01, ***P<0.001.

**Figure S4.** TET3 promotes the lipogenic program and rapid cancer growth independent of its enzymatic activity. A) Western blot showing *TET3* overexpression in *TET3*-knockout PANC-1 cells transduced with doxycycline-inducible *TET3* cDNA, treated with or without doxycycline hyclate (1 μg/ml). B) Western blot confirmation of *TET3* expression in tumor tissues from five xenografted mice. Doxycycline (0.5 mg/ml) was administered via drinking water beginning 4 weeks post-implantation for a duration of 4 weeks. C) *In vitro* proliferation of *TET3*-knockout PANC-1 cells transduced with doxycycline-inducible wild-type *TET3*, measured by IncuCyte confluence assay (n=3), with or without doxycycline (1 μg/ml). D) DNA methylation profiles across transcription start site (TSS), gene body, and transcription end site (TES) in wild-type (WT) and *TET3*-knockout (KO) PANC-1 cells. E) Volcano plot showing differentially methylated regions (DMRs) between WT and KO PANC-1 cells. Hypermethylated and hypomethylated regions are shown in red and blue, respectively. DMRs associated with deregulated lipogenic genes are labeled in black. F) Genome browser view of DNA methylation at the *SCD* locus from whole-genome bisulfite sequencing (WGBS) in WT and KO PANC-1 cells. G) Schematic of point mutations (H1077Y, D1079A) in the catalytically inactive *TET3* mutant. H) Western blot showing expression of wild-type *TET3* (TET3^wt^) or mutant *TET3* (TET3^mut^) in *TET3*-knockout PANC-1 cells with or without doxycycline treatment (1 μg/ml). I) Lipid droplets quantification by flow cytometry using BODIPY 493/503 staining in *TET3*-knockout PANC-1 cells expressing inducible *TET3^wt^* or *TET3^mut^*, with or without doxycycline (1 μg/ml). Data are presented as mean ± SD. Statistical analysis was performed using repeated measures two-way ANOVA (C). ***P<0.001.

**Figure S5.** TET3 transcriptionally represses *GATA6* through histone deacetylation. A) RT-qPCR analysis of *GATA6* mRNA levels in wild-type (WT) and *TET3*-knockout (KO) PANC-1 cells (n=3). B) Association between pathological differentiation status and *TET3* expression in TCGA-PAAD patients. Patients were grouped into TET3^high^ (top 25^th^ percentile, n=44) and TET3^low^ (bottom 25^th^ percentile, n=44) based on *TET3* mRNA expression. C) UMAP plot showing clustering of distinct cell types in PDAC tumors (GSE212966; n=6). D) Comparison of *GATA6* expression between TET3-positive and TET3-negative epithelial cells in PDAC tumors, identified in (C). E) Western blot analysis of GATA6 protein levels in *TET3*-knockout PANC-1 cells transduced with doxycycline-inducible wild-type *TET3* (TET3^wt^) or catalytically inactive *TET3* mutant (TET3^mut^), treated with or without doxycycline (1 μg/ml). F) Genome browser view of DNA methylation at the *GATA6* locus in WT and KO PANC-1 cells, based on whole-genome bisulfite sequencing. G) Schematic of ChIP-qPCR primer binding sites at the *GATA6* locus. H) RT-qPCR analysis of *GATA6* mRNA expression in WT PANC-1 cells treated with histone demethylase inhibitor Panobinostat (0, 20, 40 nM) for 24 or 48 hours (n=3). I) Western blot of GATA6 protein levels in WT PANC-1 cells treated with Panobinostat (0, 20, 40 nM) for 24 hours. Data are presented as mean ± SD. Statistical significance was determined by two-tailed unpaired t-test (A), Pearson's chi-squared test (B), two-tailed Wilcoxon test (D), or one-way ANOVA (H). **P<0.01, ***P<0.001.

**Figure S6.** GATA6 suppresses lipogenic gene expression and tumor growth. A) Tumor growth curves of nude mice subcutaneously transplanted with wild-type (WT, n=5), *TET3*-knockout (KO, n=5), or *TET3*/*GATA6* double-knockout (KO-sgGATA6, n=5) PANC-1 cells. B) Cell viability of WT PANC-1 cells treated with gemcitabine (1 μM), SAHA (5 μM), or Erastin (1 μM) for 24 or 48 hours (n=3). Data are shown as mean ± SD. Statistical significance was assessed using repeated measures two-way ANOVA (A). *P<0.05, ***P<0.001.

**Figure S7.** TET3 promotes invasive pancreatic cancer via activation of the TGF-β signaling pathway. A) UMAP plot showing clustering of distinct cell types in PDAC patients (GSE197177, n=7). B) Relative *TET3* expression in epithelial cells, identified in (A), from primary PDAC (n=3) and hepatic metastases (n=4) (GSE197177). C) Representative images and quantification of transwell invasion and migration assays in wild-type (WT) and *TET3*-knockout (KO) SW1990 cells (n=3). D) Transwell invasion and migration assays in *TET3*-knockout PANC-1 cells transduced with doxycycline-inducible *TET3* cDNA, treated with or without doxycycline (1 μg/ml) (n=3). E) Western blot analysis of EMT markers (E-cadherin, N-cadherin, and vimentin) in WT and KO SW1990 cells. F) Volcano plot of differentially accessible regions (DARs) between WT and KO PANC-1 cells. Hyper-accessible and hypo-accessible regions are shown in red and blue, respectively. DARs associated with *TGFB2* are labeled in black. G) Western blot showing *TET3* knockout efficiency in CFPAC-1 cells. H) Western blot confirming *SMAD4* knockout in WT and *TET3*-knockout (KO) PANC-1 cells. I) Representative images and quantification of transwell invasion assays in WT and KO PANC-1 cells transduced with control sgRNA (sgCtrl) or SMAD4-targeting sgRNA (sgSMAD4) (n=3). Scale bars = 200 μm. Data are presented as mean ± SD. Statistical significance was assessed by two-tailed Wilcoxon test (B), two-tailed unpaired t-test (C, D), or one-way ANOVA (I). *P<0.05, **P<0.01, ***P<0.001.

**Figure S1**

**
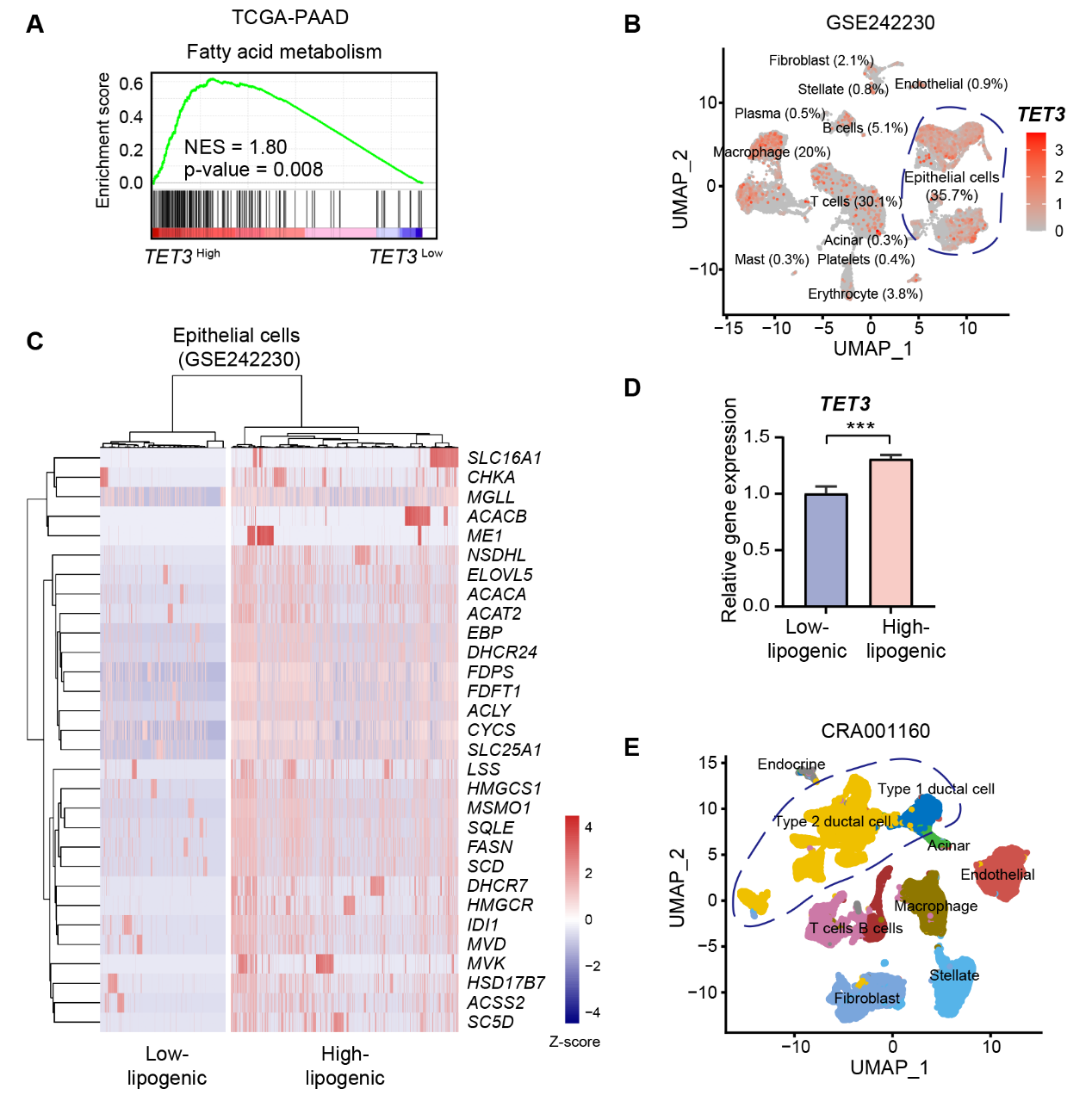
**

**Figure S2**

**
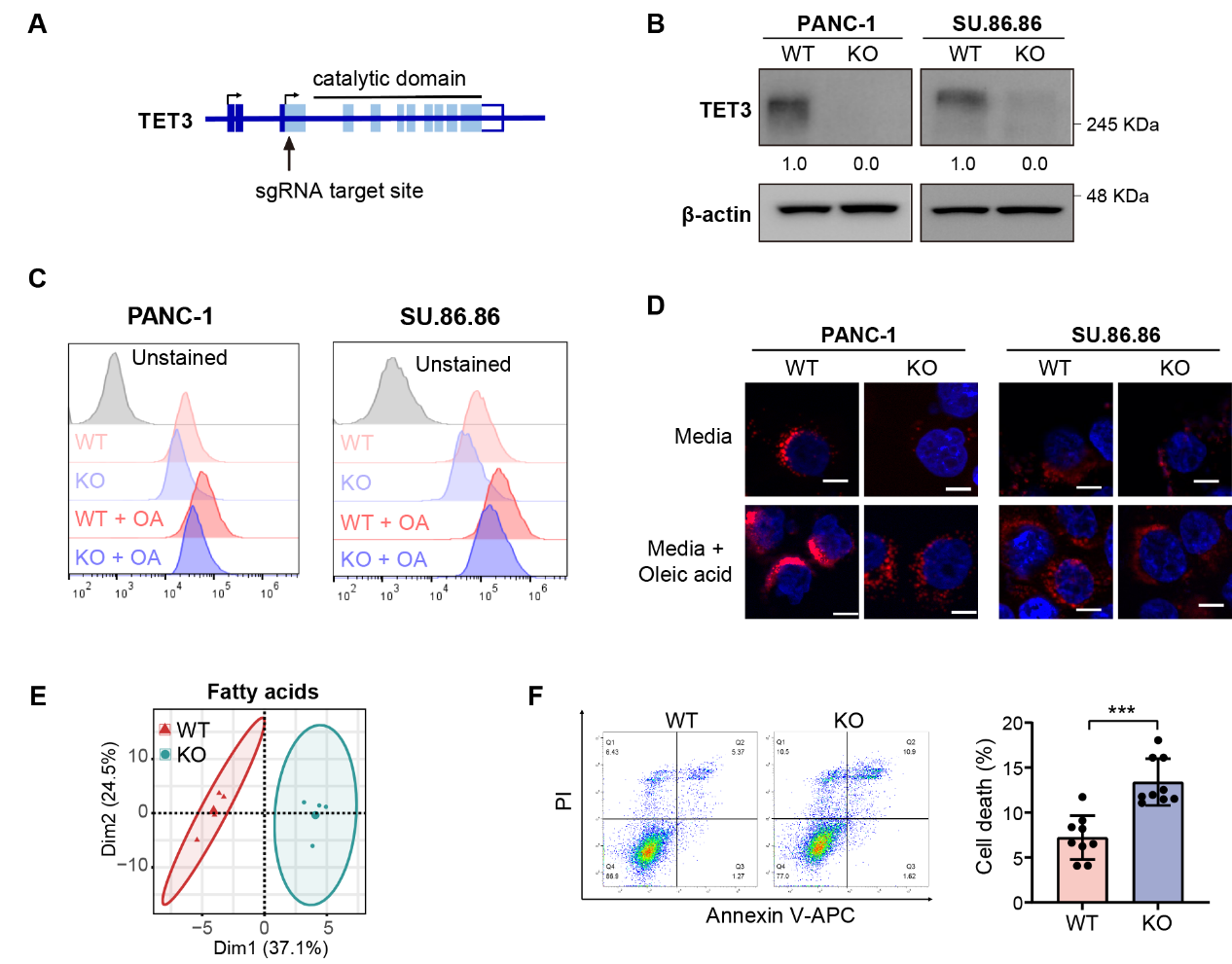
**

**Figure S3**

**
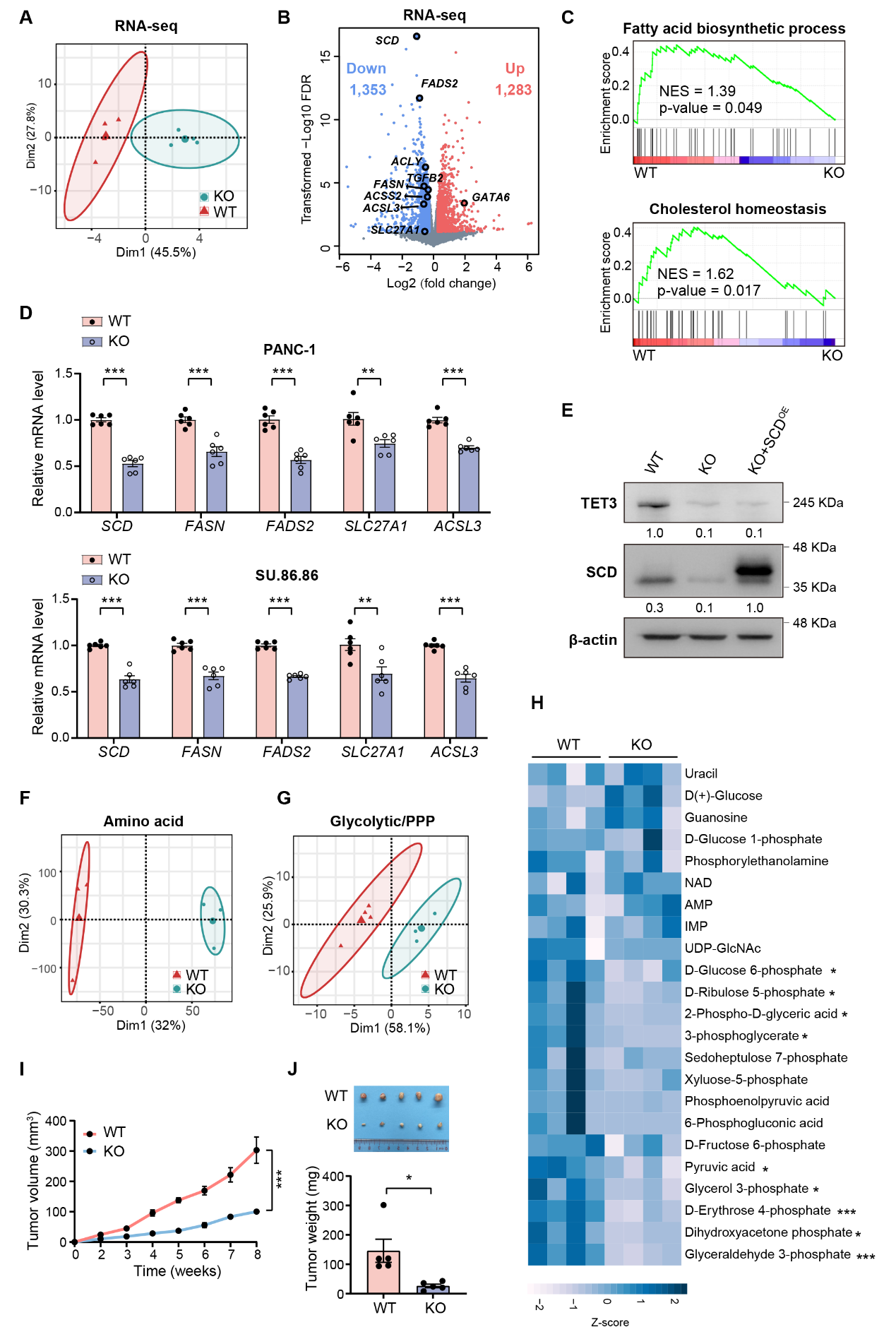
**

**Figure S4**

**
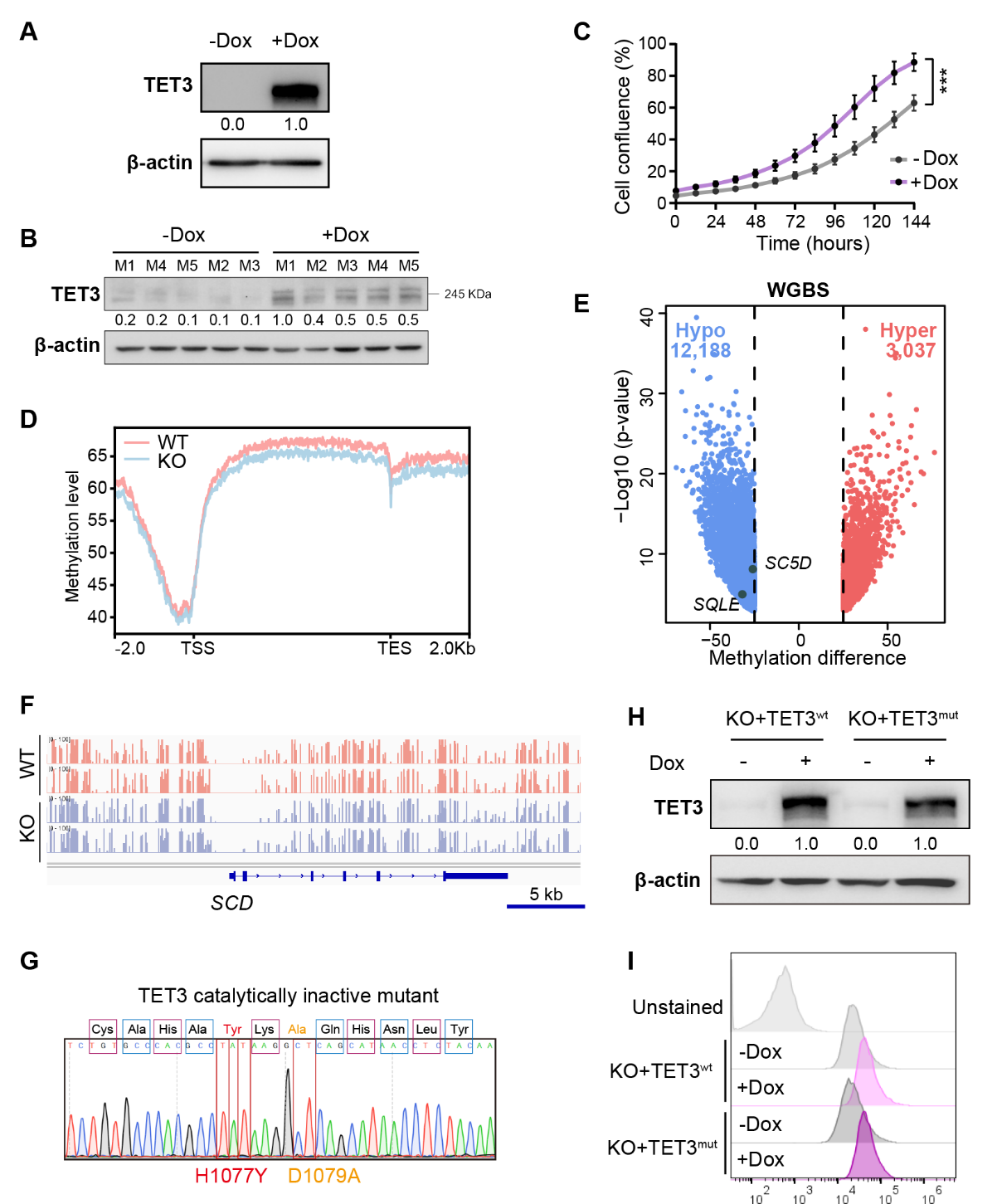
**

**Figure S5**

**
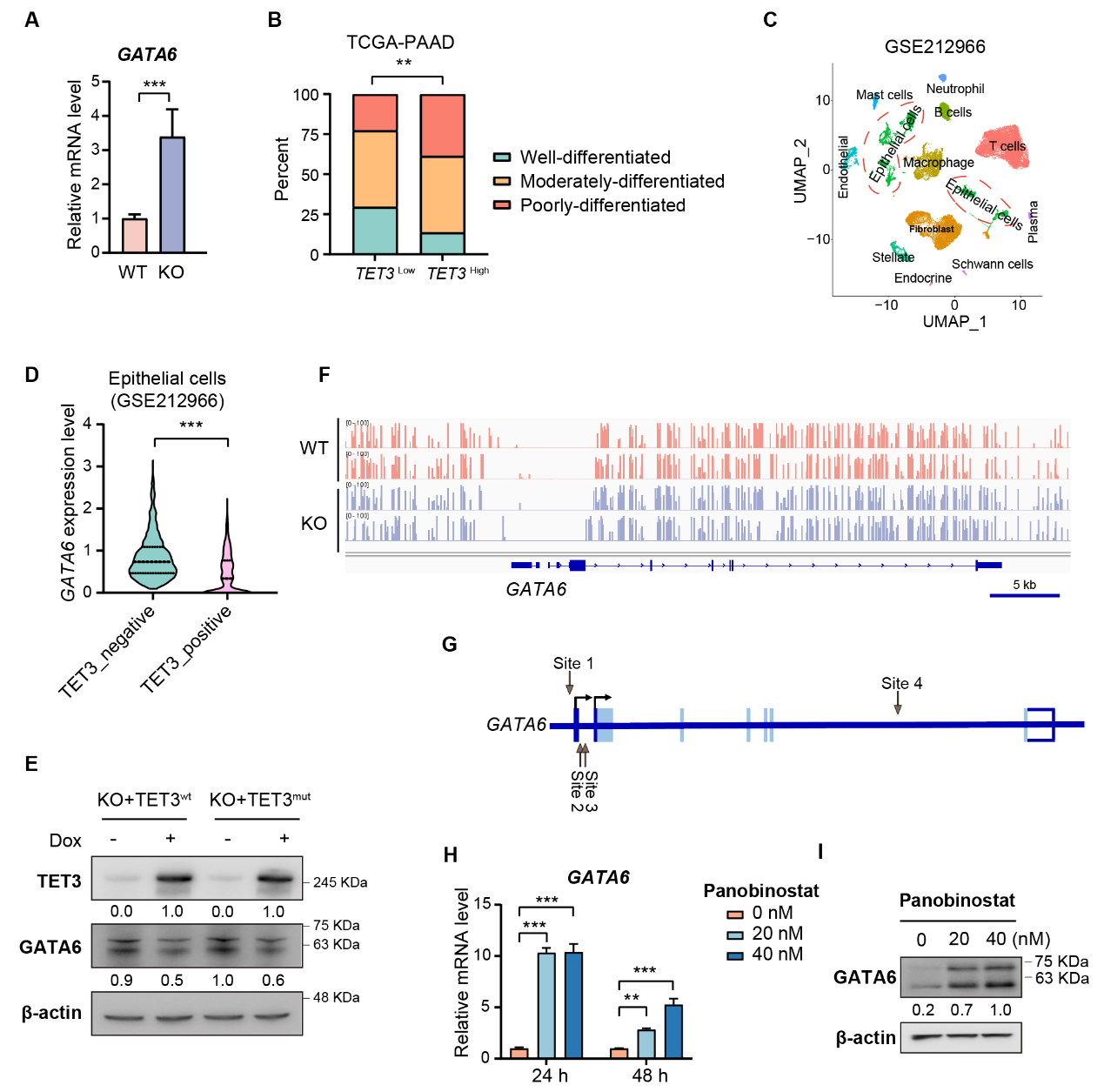
**

**Supplementary Figure S6**

**
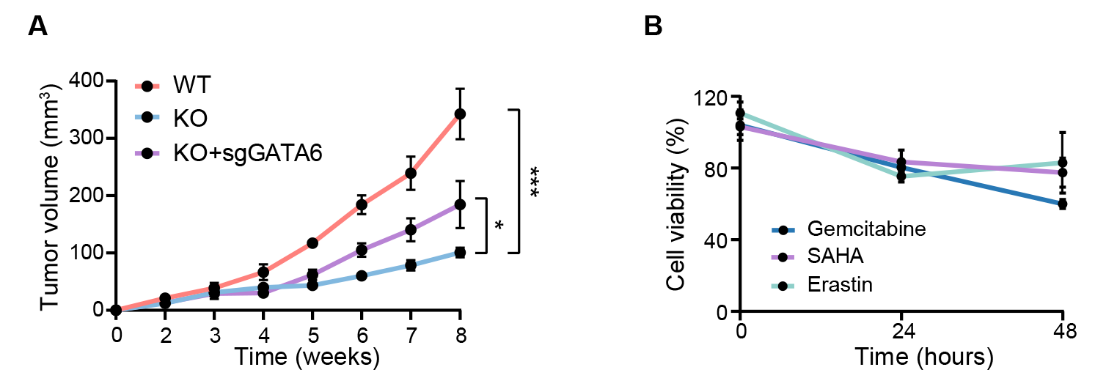
**

**Figure S7**

**
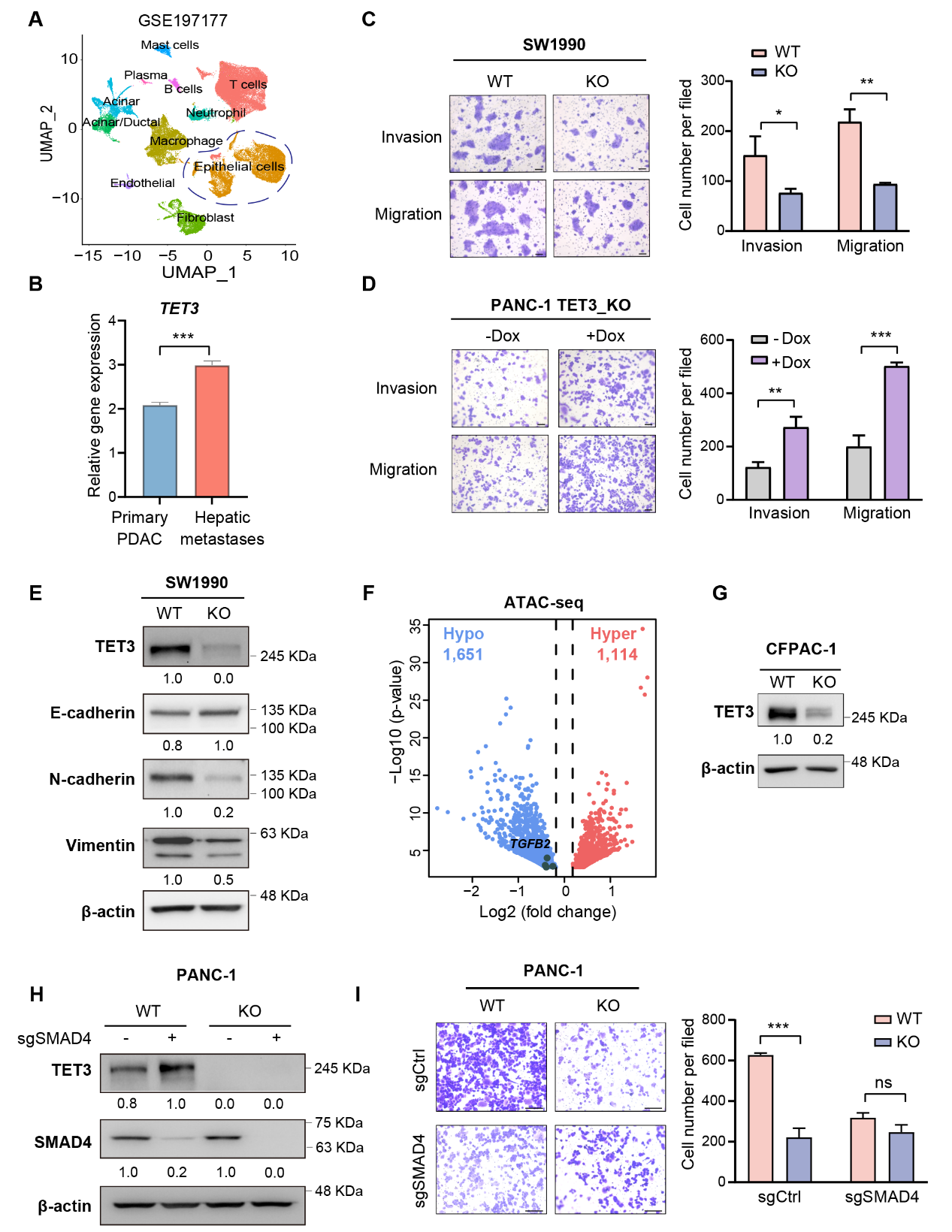
**

**Supplementary Tables**

**Supplementary Table 4. Primers for qPCR**

| Gene | Forward | Reverse |
| --- | --- | --- |
| SCD | CCTGGTTTCACTTGGAGCTGTG | TGTGGTGAAGTTGATGTGCCAGC |
| FASN | TTCTACGGCTCCACGCTCTTCC | GAAGAGTCTTCGTCAGCCAGGA |
| FADS2 | TGCAACGTGGAGCAGTCCTTCT | GGCACATAGAGACTTCACCAGC |
| ACSL3 | CTTTCTCACGGATGCCGCATTG | CTGCTGCCATCAGTGTTGGTTTC |
| SLC27A1 | TGACAGTCGTCCTCCGCAAGAA | CTTCAGCAGGTAGCGGCAGATC |
| GATA6 | GCCACTACCTGTGCAACGCCT | CAATCCAAGCCGCCGTGATGAA |
| TBP | TGTGCACAGGAGCCAAGAGT | ATTTTCTTGCTGCCAGTCTGG |
| ChIP-1 | CCTCCTCCTCTTCACCTCCA | CACCGAGCCCTAAACAAACAG |
| ChIP-2 | GCGGACCAACTTCTAGTCTCTT | TTCTCTGCCTGCCTAACTACC |
| ChIP-3 | CGACGGTGTTTCCCTCCTT | ACTCGGAGATAAGGCTGCG |
| ChIP-4 | GGAGTTGACCAATCATCACAGT | CCTGGCACATAGGGAGCAT |
| ChIP-neg | ATGCCAAGTGAAATAGCCAGTC | CCACCATCCATCTCCAGAAGT |

**Supplementary Table 5. Antibodies**

| Protein | Company | Catalog |
| --- | --- | --- |
| TET3 | Cell Signaling Technology | 85016 |
| GATA6 | Cell Signaling Technology | 5851 |
| E-cadherin | Cell Signaling Technology | 3195 |
| N-cadherin | Cell Signaling Technology | 13116 |
| Vimentin | Cell Signaling Technology | 5741 |
| Phospho-SMAD2/3 | Cell Signaling Technology | 8828 |
| SMAD2/3 | Cell Signaling Technology | 3102 |
| SMAD4 | Cell Signaling Technology | 46535 |
| TGF-β2 | Proteintech | 19999-1-AP |
| H3K27ac | Activ Motif | 39133 |
| V5 | Cell Signaling Technology | 13202 |
| HDAC1 | Cell Signaling Technology | 2062 |
| HDAC2 | Cell Signaling Technology | 2540 |
| SCD | Proteintech | 28678-1-AP |
| β-actin | Santa Cruz | sc47778 |

**Supplementary Table 6. Reagents and compounds**

| Name | Formula | Company | Catalog | Stock in DMSO |
| --- | --- | --- | --- | --- |
| CAY10566 | C_18_H_17_ClFN_5_O_2_ | MedChemExpress | HY-15823 | 10 mM |
| Orlistat | C_29_H_53_NO_5_ | MedChemExpress | HY-B0218 | 100 mM |
| Gemcitabine | C_9_H_11_F_2_N_3_O_4_ | MedChemExpress | HY-17026 | 100 mM |
| SAHA | C_14_H_20_N_2_O_3_ | MedChemExpress | HY-10221 | 100 mM |
| Panobinostat | C_21_H_23_N_3_O_2_ | Activ Motif | 14045 | 100 mM |
| Erastin | C_30_H_31_ClN_4_O_4_ | TargetMol | T1765 | 20 mM |
